# Thermoelectric Modulation of Neat Ti_3_C_2_T_x_ MXenes by Finely Regulating the Stacking of Nanosheets

**DOI:** 10.1007/s40820-024-01594-z

**Published:** 2024-12-26

**Authors:** Junhui Tang, Renyang Zhu, Ya-Hsin Pai, Yan Zhao, Chen Xu, Ziqi Liang

**Affiliations:** 1 Department of Materials Science, Fudan University, Shanghai, 200433 People’s Republic of China; 2 Spine Center, Department of Orthopedics, Shanghai Changzheng Hospital, Naval Medical University, Shanghai, 200003 People’s Republic of China

**Keywords:** MXene, Nanosheet stacking, Electrical conductivity, Seebeck coefficient, Thermoelectrics

## Abstract

Emerging two-dimensional MXenes have been extensively studied in a wide range of fields thanks to their superior electrical and hydrophilic attributes as well as excellent chemical stability and mechanical flexibility. Among them, the ultrahigh electrical conductivity (*σ*) and tunable band structures of benchmark Ti_3_C_2_T_x_ MXene demonstrate its good potential as thermoelectric (TE) materials. However, both the large variation of *σ* reported in the literature and the intrinsically low Seebeck coefficient (*S*) hinder the practical applications. Herein, this study has for the first time systematically investigated the TE properties of neat Ti_3_C_2_T_x_ films, which are finely modulated by exploiting different dispersing solvents, controlling nanosheet sizes and constructing composites. First, deionized water is found to be superior for obtaining closely packed MXene sheets relative to other polar solvents. Second, a simultaneous increase in both *S* and *σ* is realized via elevating centrifugal speed on MXene aqueous suspensions to obtain small-sized nanosheets, thus yielding an ultrahigh power factor up to ~ 156 μW m^−1^ K^−2^. Third, *S* is significantly enhanced yet accompanied by a reduction in *σ* when constructing MXene-based nanocomposites, the latter of which is originated from the damage to the intimate stackings of MXene nanosheets. Together, a correlation between the TE properties of neat Ti_3_C_2_T_x_ films and the stacking of nanosheets is elucidated, which would stimulate further exploration of MXene TEs.

**Supplementary Information:**

The online version contains supplementary material available at 10.1007/s40820-024-01594-z.

## Highlights

- Investigation of dispersing solvents on processing Ti_3_C_2_T_x_ thin films revealed that deionized water is superior to realize tight stacking and high orientation of MXene nanosheets.
- A simultaneous elevation of Seebeck coefficient and electrical conductivity of neat Ti_3_C_2_T_x_ films is achieved by increasing the centrifugal speed of MXene aqueous suspensions due to the energy filtering effect.
- Further construction of Ti_3_C_2_T_x_ nanocomposites significantly strengthens Seebeck coefficient yet disrupts the stacking of MXene nanosheets.

**Supplementary Information:**

The online version contains supplementary material available at 10.1007/s40820-024-01594-z.

## Graphical abstract

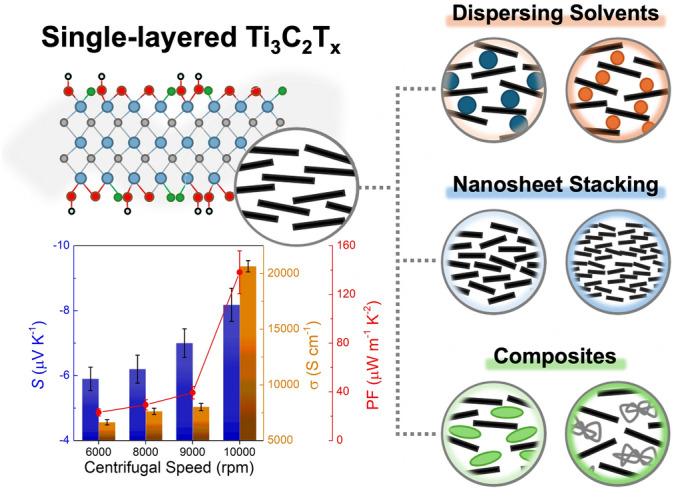

## Introduction

Since the initial discovery in 2011 [1], transition metal carbides and nitrides (MXenes), as an emerging class of two-dimensional (2D) materials, have become increasingly hotspots thanks to their superior electrical, optical and hydrophilic attributes as well as excellent chemical stability and mechanical flexibility [2–4]. MXenes are nominally represented by a formula of M_n+1_X_n_T_x_ (*n* = 1, 2 or 3) where M is an early transition metal (such as Sc, Ti, V, Mo or Nb), X represents carbon or nitrogen, and T denotes surface terminal groups such as −O, −OH and −F [5, 6]. In principle, MXenes were synthesized by selective etching that removed the A element (such as Al, Si, Ge or Sn) from the parent carbide or nitride compounds [7]. Hitherto, over 30 kinds of MXenes have been successfully synthesized, among which Ti_3_C_2_T_x_ MXene remains the best studied, holding great prospects for energy storage [8], electromagnetic interference shielding [9, 10], sensor [11, 12], catalysis [13] and fire warning [14], all benefiting from the diverse merits in physical, chemical and mechanical properties [15]. Electrical conductivity (*σ*) of Ti_3_C_2_T_x_ MXene films can be readily tuned; for instance, an outstanding *σ* as high as ~ 15,000 S cm^−1^ was realized in MXene films containing highly aligned large Ti_3_C_2_T_x_ flakes by blade-coating, whereas the *σ* of MXene films obtained by HF etching was only ~ 1,500 S cm^–1^ [16, 17]. Moreover, the surface functional groups also affected the *σ* of Ti_3_C_2_T_x_ sheets, with −O-terminated Ti_3_C_2_T_x_ usually exhibiting higher *σ* than that of −F or −OH-terminated ones [18].

Thermoelectric (TE) materials, as environmentally friendly materials that can directly convert waste heat into electricity, have received increasing attention in the framework of sustainable deployment [19–21]. The TE performance of materials is evaluated by the dimensionless figure of merit (*zT*), defined as *zT* = *S*^2^*σT*/*κ* whereby *S*, *T* and *κ* are the Seebeck coefficient, temperature and thermal conductivity, respectively. A desirable *zT* value is ideally attained by a synergy of high *σ*, high *S* and low *κ*, in spite of their intimate couplings [22, 23]. It should be stressed that the *S* is closely correlated to the electronic band structure of the materials, which is more chemically tailorable in Ti_3_C_2_T_x_ than those massively studied 2D materials—MoS_2_ [24], SnSe [25] and black phosphorus [26], while presenting excellent *σ* comparable to graphene [27]. These merits have endowed Ti_3_C_2_T_x_ MXene with a promising candidate for TE applications.

Most reported studies were focused on TE composites with Ti_3_C_2_T_x_ MXene that was exploited as one single component in conjunction with carbon nanotubes [28, 29], polymers [30, 31] and inorganic TE materials [32–41], for example, the slight addition of Ti_3_C_2_T_x_ into single-walled carbon nanotubes (SWCNTs) [28] and poly(3,4-ethylenedioxythiophene):poly(styrenesulfonate) (PEDOT:PSS) [30], respectively, to significantly enhance *S* by providing electrons to compensate a small number of holes while preserving the p-type nature of matrices. Similarly, Ti_3_C_2_T_x_ (less than 5 wt% and even an ultralow content of 0.1 wt%) was blended with inorganic TE materials, with a prime goal of increasing carrier concentration (*n*) via the electron transfer process between two components [32–36], or enhancing carrier mobility (*μ*) by delocalization of electrons [37], as well as a delicate reinforcement of both *n* and *μ* [38]. Moreover, the lattice thermal conductivity (*κ*_L_) was drastically reduced by boosting interfacial phonon scattering via the creation of abundant grain boundaries through Ti_3_C_2_T_x_ inclusion in the TE composites [32, 35, 37, 38, 39, 40].

In stark contrast, only a tiny portion of the researches were solely focused on the TE properties of neat MXene films. Among them, theoretical calculations on the TE performance of MXenes were extensively studied, including Sc_2_CT_2_, Ti_2_CT_2_, V_2_CT_2_, Ta_2_CT_2_, Mo_2_CT_2_, Y_2_CT_2_, where T = −O, −F, −OH [42–47]. A champion *zT* value was predicted to be approximately 3 in p-type Sc_2_TiC_2_(OH)_2_ [48], which has unfortunately far yet to be validated by the experimental results. On the one hand, the carrier concentration of MXenes was fixed as a parameter, which is not necessarily possible to achieve in MXenes due to its difficulty in tuning *n* as metallic materials. On the other hand, the surface functional group was also set as merely −O, −F, or −OH, which is a typical combination of all these functional groups in practical materials. By contrast, only a few studies were focused on the experimental investigation of the TE properties of neat MXenes. Both Mo_2_CT_x_ and Nb_2_CT_x_ were found to be transformed from p-type to n-type upon high-temperature thermal annealing [49, 50]. An intercalation of K^+^ into Ti_3_C_2_T_x_ films notably improved *S* by threefold and also maintained the originally high *σ* = 1,652 S cm^−1^, resulting in an optimal power factor (PF) of 44.98 μW m^−1^ K^−2^ at room temperature [51]. However, the literature reported *σ* values of benchmark Ti_3_C_2_T_x_ thin films varied enormously with each other [3, 16, 17, 51], for which the key impacting factors remain highly elusive.

As 2D materials, the contacts between MXene nanosheets play a crucial role in impacting charge transport behaviors of MXene films. Given that single-layered (SL)-Ti_3_C_2_T_x_ MXenes were obtained by exfoliating multi-layered (ML)-MXenes, the dispersing solvents were generally intercalated into the MXene sheets, thereby greatly affecting the stacking distances and the contacting degrees. We therefore firstly investigated in this work the dispersing solvents for MXene films and found deionized water to be more favored to achieve tightly stacked MXene sheets than other polar solvents. Second, the centrifugal speed of SL-MXene suspensions was examined, which also largely affected the stackings of MXene sheets. It was revealed that higher speeds aided in the acquisition of smaller-sized nanosheets, which is conducive to the extremely close stacking of MXene nanosheets with high orientation along the in-plane direction and led to an impressively high *σ* of ~ 20,000 S cm^−1^. Meanwhile, the *S* was raised, which breaks the coupled relation between *S* and *σ*, leading to a champion PF of ~ 156 μW m^−1^ K^−2^ at room temperature. Third, the *S* of Ti_3_C_2_T_x_ MXenes remained ultralow owing to their intrinsically metal-like behaviors and high carrier concentration, which immensely hinders potential TE applications. Thus, nanocomposites based on MXene and sodium deoxycholate or single-walled carbon nanotubes were fabricated and unveiled to be effective in enhancing *S* yet dramatically diminishing *σ*, stressing the importance of maintaining the tight stacking of MXene nanosheets.

## Experimental Section

### Materials

All chemicals and reagents were used as received and without any further purification, including Ti_3_AlC_2_ (Jilin 11 Technology Co., Ltd), sodium deoxycholate (Beijing InnoChem Science & Technology Co., Ltd), single-walled carbon nanotubes (Shenzhen Nanotech Port Co., Ltd) and lithium fluoride (Shanghai Aladdin Biochemical Technology Co., Ltd) as well as *N*,*N*-dimethylformamide (≥ 99.5%), ethanol (≥ 99.5%) and hydrochloric acid (36.0–38.0 wt%), all of which were purchased from Sinopharm Chemical Reagent Co., Ltd.

### Synthesis of MXene Thin Films

**Fig. 1:**
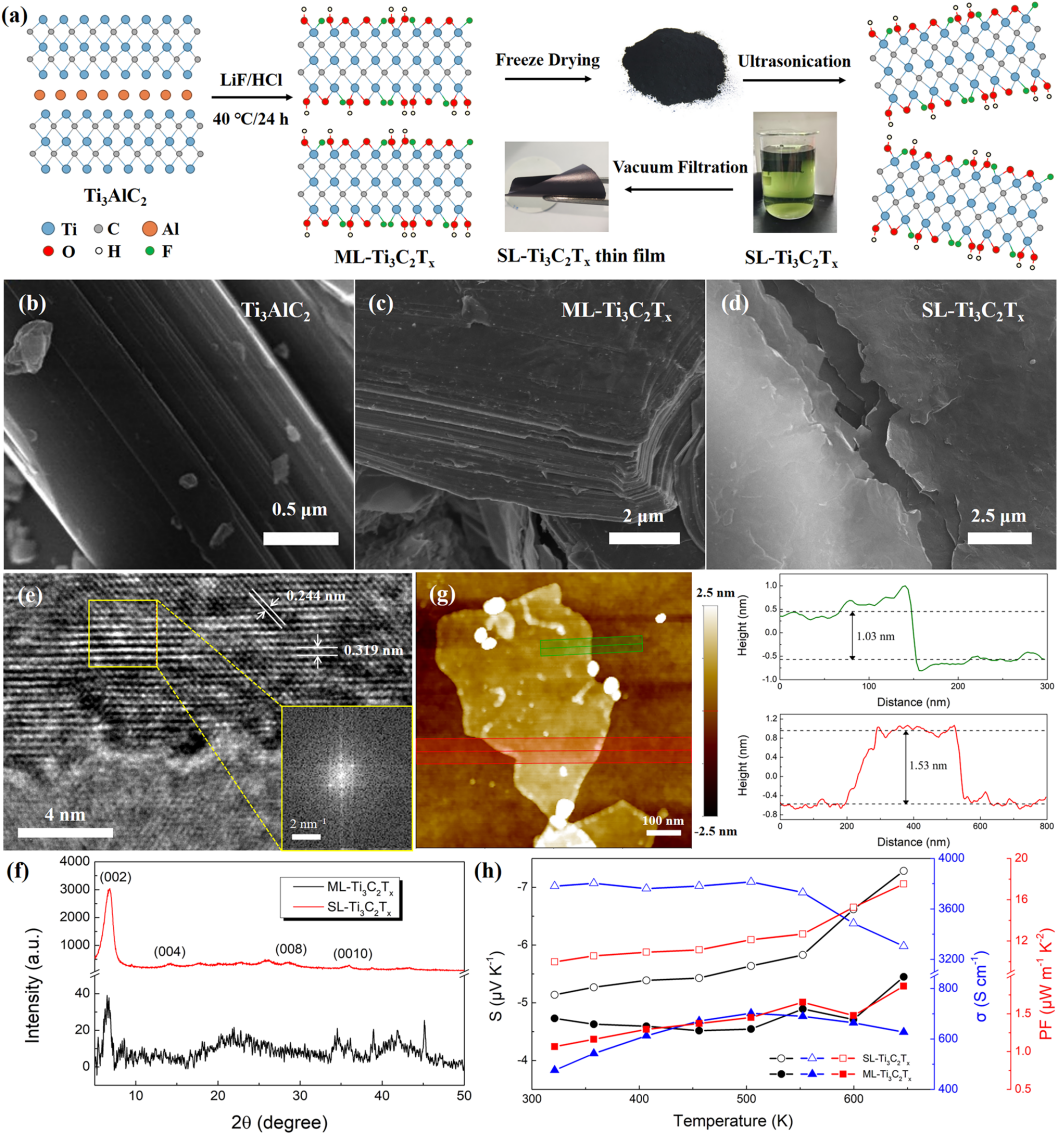
Synthesis and characterization of Ti_3_C_2_T_x_ MXene films. **a** Synthetic procedures of single-layered (SL)-Ti_3_C_2_T_x_ MXene from Ti_3_AlC_2_ MAX powders. Characterization of Ti_3_AlC_2_ powders, ML-Ti_3_C_2_T_x_ powders, and SL-Ti_3_C_2_T_x_ thin film: **b**–**d** FE-SEM images, **e** HR-TEM image, **f** XRD patterns, and **g** TP-AFM image. **h** Temperature-dependent thermoelectric properties of ML- and SL-Ti_3_C_2_T_x_ thin films during the heating process.

Ti_3_C_2_T_x_ MXene was chemically synthesized by etching Ti_3_AlC_2_ MAX phase with LiF and HCl as shown in Fig. 1a, and the specific process was as follows. At first, a mixture of LiF (1 g), deionized (DI)-water (5 mL) and hydrochloric acid (15 mL, 12 M) was added into a Teflon reaction flask, after which Ti_3_AlC_2_ powders were slowly added into the flask and stirred at 40 °C in water bath for 24 h. After etching, the suspension was divided into three centrifuge tubes, and then DI-water was added to equalize the mass. Then the dispersions were centrifuged at a rate of 3,500 rpm, followed by pouring out the supernatant. This process was repeated until the pH value increased to more than 6. The remaining suspensions were further vacuum filtered, and the obtained precipitates were freeze dried to obtain multi-layered (ML)-Ti_3_C_2_T_x_ MXene powders.

The ML-Ti_3_C_2_T_x_ powders were weighed and dispersed in DMF and EtOH solvents to prepare two types of MXene dispersions at specific concentrations. However, to obtain the single-layered (SL)-Ti_3_C_2_T_x_, the ML-Ti_3_C_2_T_x_ powders were dispersed in DI-water and further sonicated in an ice bath for 3 h. The above dispersions were centrifuged at 3500 rpm and the supernatant was collected as SL-Ti_3_C_2_T_x_ dispersions, while the remaining precipitates could be reused for SL-MXene preparation. The SL-Ti_3_C_2_T_x_ thin film was finally acquired upon vacuum filtration.

The SL-MXenes with different distribution of nanosheet sizes were achieved by vigorously hand-shaking and centrifuging the above-ultrasonicated dispersions at the rates of 10,000, 9000, 8000, and 6000 rpm, respectively. Note that the relatively slow centrifugation process was performed on the suspensions composed of precipitates that remained after rapid centrifugation with DI-water. To determine the concentration of SL-MXene dispersions, a specific volume of suspension is vacuum filtered onto a filter paper with a known mass, and then the filter paper was weighed after thorough drying to calculate the concentration. Sodium deoxycholate (NaDC) and single-walled carbon nanotubes (SWCNTs) were separately added into the SL-Ti_3_C_2_T_x_ suspensions of known concentration. The nanocomposite films were obtained by vacuum filtering the sonicated MXene suspensions with NaDC or SWCNTs, respectively.

### Characterization and Measurements

A four-probe technique was used to measure the electrical conductivity on a multimeter (Keithley 2010) and a source meter (Keithley 2400). The Seebeck coefficient was measured by heating one side of the samples with a resistor block while simultaneously measuring the generated temperature gradient (ΔT) and thermoelectric voltage (ΔV). Field-emission scanning electron microscopy (FE-SEM) images were acquired on a JEOL JSM-6701F at an accelerating voltage of up to 30 kV. High-resolution transmission electron microscopy (HR-TEM) imaging was performed on a JEM-2100 (JEOL Ltd.) at an accelerating voltage of 200 kV, with the samples prepared by dropping dilute SL-MXene dispersions on a copper grid. X-ray diffraction (XRD) was carried out on a Bruker AX D8 Advance diffractometer with nickel-filtered Cu Ka radiation (λ = 1.5406 Å). Atomic force microscope (AFM) images were collected using a Dimension Edge (Bruker Nano Inc.). For AFM imaging, the samples were prepared by spin-coating dilute SL-MXene suspensions on the Si substrate, and the measurement was taken using NX10 microscope from Park Systems Co. Ltd. in tapping mode. Ultraviolet–visible–near-infrared (UV–vis–NIR) absorption spectra were measured on HITACHI U-4100 Spectrophotometer. For grazing incidence wide-angle X-ray scattering (GIWAXS) measurements, the 2D diffraction patterns were acquired by beamline BL14B1 at Shanghai Synchrotron Radiation Facility (SSRF) with an X-ray source of 10 keV and an incident angle of 0.2°. Thermogravimetric analysis (TGA) was conducted on a TGA8000 (PE, USA) with a heating rate of 10 K min^−1^ under N_2_ atmosphere. X-ray photoelectron spectroscopy (XPS) measurements were taken on an X-ray photoemission spectroscope (PHI5300) using monochromatic Al Kα X-rays with the pass energy of 40 eV; all the peaks were calibrated by C 1*s*. The dispersion of MXene nanosheet size was characterized by a nanoparticle size and Zeta potential analyzer (Malvern Zetasizer Nano ZS90). Ultraviolet photoelectron spectroscopy (UPS) of MXene films was conducted by Thermo Fisher ESCALAB XI+ spectrometer. Carrier concentration and Hall mobility were measured by a Nanometrics HL5500 Hall system using the van der Pauw technique at room temperature under a magnetic field of 0.32 T.

## Results and Discussion

### High-Quality Ti_3_C_2_T_x_ MXene via Freeze Drying

Different from those reported works on the synthesis of Ti_3_C_2_T_x_ MXene [3, 4], the freeze process [52, 53] was exploited in this work, instead of conventional drying under a relatively high temperature, to prevent oxidation of Ti_3_C_2_T_x_ nanosheets and to acquire high-quality Ti_3_C_2_T_x_ suspensions and corresponding thin films. To demonstrate the successful synthesis of both ML- and SL-Ti_3_C_2_T_x_ MXenes, field-emission scanning electron microscopy (FE-SEM) was used to characterize the microstructures of Ti_3_AlC_2_ MAX powders as well as ML- and SL-MXenes as shown in Fig. 1b–d. ML-MXene shows an accordion structure, while SL-MXene displays an ordered and compact structure, which may be beneficial to maintain a high carrier mobility. Low-magnification transmission electron microscopy (TEM) image of SL-Ti_3_C_2_T_x_ and the corresponding energy-dispersive X-ray spectroscopy (EDS) elemental mapping images are shown in Fig. S1, which exhibit the uniform distribution of Ti, C, O and F elements in MXenes. As shown in the high-resolution (HR)-TEM image of the SL-MXene (Fig. 1e), the distinct lattice fringes of 0.244 and 0.319 nm are clearly observed, and the former interplanar spacing corresponds to the (*103*) plane of Ti_3_C_2_T_x_ MXene [54, 55]. All these evidences indicate that Ti_3_C_2_T_x_ is successfully synthesized.

Structural variation of the MXene films was studied by X-ray diffraction (XRD) patterns (Fig. 1f). A relatively weak peak of (*002*) around 6.56° for ML-Ti_3_C_2_T_x_ film reveals an interlayer spacing of 1.35 nm [56, 57]. By contrast, the sharp (*002*) peak in SL-Ti_3_C_2_T_x_ film implicitly shifts to 6.67° (1.33 nm), suggesting that the interlayer distance remains relatively unaltered during the transition from ML-MXene to SL-MXene. The significant strengthening of the (*002*) peak implies that the stacking between nanosheets might be more ordered in SL-MXene, resulting in a comparatively high-intensity crystallization signal. To further validate the successful exfoliation of ML-Ti_3_C_2_T_x_ into SL-Ti_3_C_2_T_x_, tapping-mode atomic force microscopy (TP-AFM) was employed to observe the spin-coated thin film by dilute SL-Ti_3_C_2_T_x_ suspensions. As shown in Fig. 1g, a relatively flat sheet with a size of several hundred nanometers is observed, the thickness of which was characterized by the green and red height curves showing the cross-sectional plots of MXene sheet. Since the interlayer spacing of SL-Ti_3_C_2_T_x_, which reflects the distance between the center of two adjacent nanosheets, is determined to be 1.33 nm by XRD results, the thickness of MXene sheets should be around 1.30 nm by considering the gap between nanosheets. Thus, the thickness of 1.53 nm is a direct evidence of the successful acquisition of SL-Ti_3_C_2_T_x_ MXene and the occurrence of thickness of 1.03 nm is presumably due to the detachment of surface functional groups. Moreover, several pieces of Ti_3_C_2_T_x_ MXenes with micrometer-sized diameters can be observed in a same TP-AFM image as shown in Fig. S2, which can further prove the successful synthesis of SL-Ti_3_C_2_T_x_.

Temperature-dependent TE properties of ML- and SL-Ti_3_C_2_T_x_ thin films are displayed in Figs. 1h and S3. The trend of *σ* with respect to temperature differs slightly in both films. In SL-MXene film, the *σ* is insensitive to temperature below 500 K, whereas in ML-MXene films, the *σ* continuously increases within this temperature range. Yet the *σ* of both films continues to drop rapidly with the increase of temperature up to 650 K. Since Ti_3_C_2_T_x_ MXene presents metallic characteristics [49, 50], the *σ* typically drops gradually with the elevation of temperature. However, the loss of intercalated water molecules in the MXene film during the heating process would lead to a decrease in the interlayer distance, which can be evidenced by the fact that the (*002*) peak of Ti_3_C_2_T_x_ film continuously shifts to higher degrees with an increasing annealing temperature as shown in Fig. S4. Moreover, the charge transport between MXene nanosheets becomes more efficient since the measured Hall mobility of Ti_3_C_2_T_x_ film undergoes a synchronous uplifting trend with the annealing temperature (see Fig. S5). Thus, these two opposing factors compete with each other and eventually result in the phenomenon that the *σ* of SL-MXene film reaches a plateau at ~ 3,800 S cm^−1^ while that of ML-MXene film steadily rises from ~ 500 to ~ 700 S cm^−1^ in the range of 300−500 K, respectively. For higher temperatures over 500 K, however, the intercalated water molecules have already been removed and the increased resistance to directional movement of electrons with elevated temperature leads to the decrease of *σ* down to 3305 and 628 S cm^−1^ in SL- and ML-Ti_3_C_2_T_x_ films at around 645 K, respectively. Meanwhile, the *S* generally shows an elevating trend with increasing temperature thanks to the magnon drag effect [58], and the similar trend is reported in other types of MXenes such as Mo_2_TiC_2_T_x_ and Mo_2_Ti_2_C_3_T_x_ [49]. Together, the PF of SL-Ti_3_C_2_T_x_ thin film is significantly strengthened from 10.0 μW m^−1^ K^−2^ at 321 K to 17.5 μW m^−1^ K^−2^ at 646 K. Since the heating process left an unrecoverable effect of removing solvent molecules on Ti_3_C_2_T_x_ thin films, the TE properties at room temperature after cooling surely cannot return to the original value. It turns out that *S* and *σ* are increased to −6.2 μV K^−1^ and 5364 S cm^−1^ in SL-MXene film, respectively, highlighting 20.2% and 41.9% enhancements with respect to the initial TE performance, jointly elevating the PF up to 20.5 μW m^−1^ K^−2^.

### Dispersing Solvents for MXene Films

**Fig. 2:**
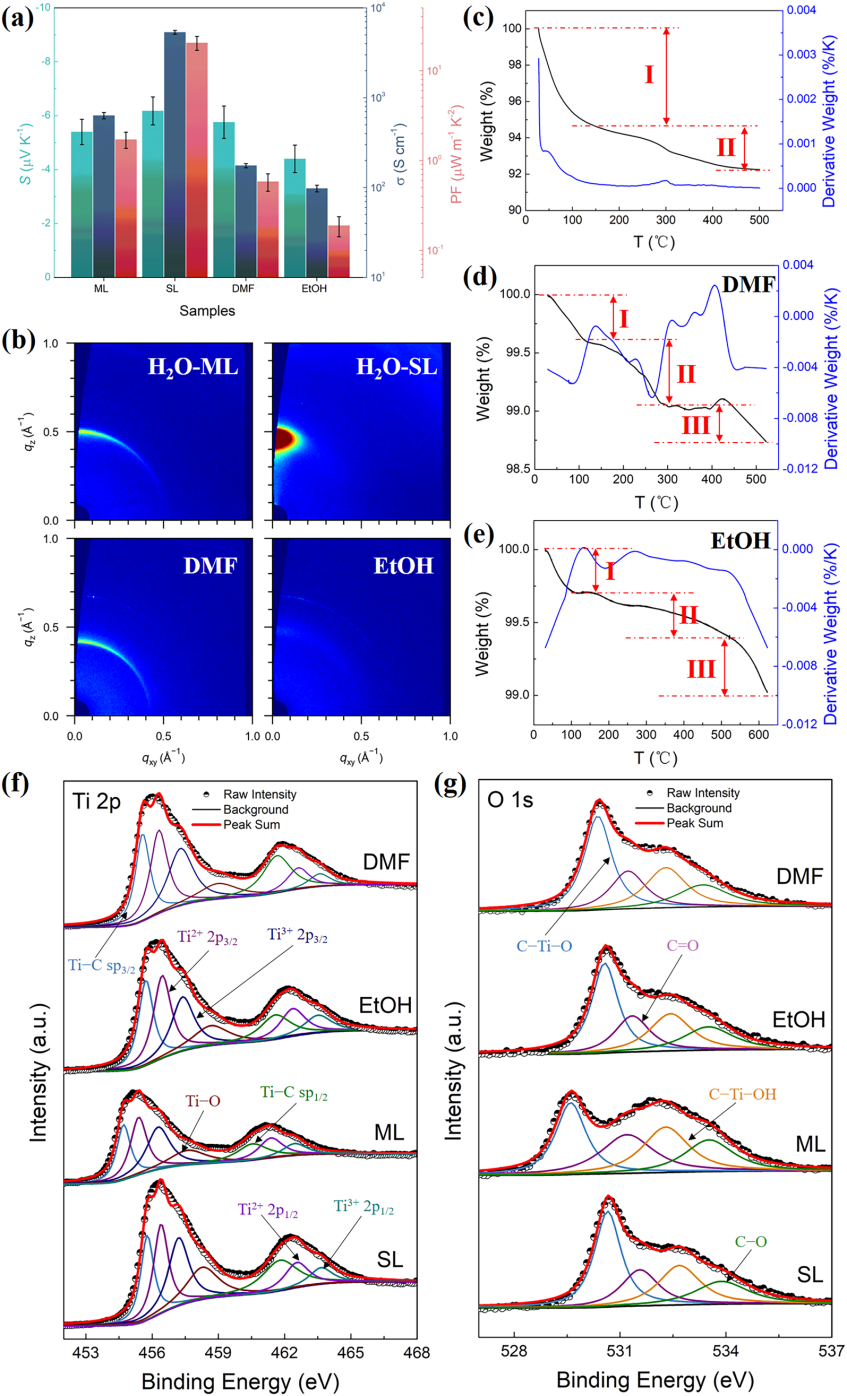
Effects of dispersing solvents on Ti_3_C_2_T_x_ MXene films. **a** Thermoelectric properties of Ti_3_C_2_T_x_ thin films obtained by processing with different dispersing solvents of H_2_O, DMF and EtOH. **b** GIWAXS profiles of ML- and SL-Ti_3_C_2_T_x_ thin films acquired by H_2_O-based suspensions as well as Ti_3_C_2_T_x_ thin films obtained by DMF- and EtOH-based suspensions. TGA results of Ti_3_C_2_T_x_ films obtained from suspensions with **c** H_2_O, **d** DMF and **e** EtOH as dispersing agents, respectively. XPS patterns of **f** Ti 2*p* and **g** O 1*s* for Ti_3_C_2_T_x_ thin films processed with various dispersing solvents.

The above studies indicated that solvent molecules may have an unignorable effect on the TE properties of MXene thin films. Therefore, the TE performance of Ti_3_C_2_T_x_ films treated with different solvents under room temperature was further investigated, and the results are shown in Fig. 2a. Note that all Ti_3_C_2_T_x_ films were thermally annealed at 400 °C for 30 min prior to their TE measurements. The SL-Ti_3_C_2_T_x_ film processed from water shows *σ* (5364 S cm^−1^) nearly two orders of magnitude higher than those processed with DMF (176 S cm^−1^) and EtOH (98 S cm^−1^). Given that the *S* of all MXene films with these dispersing solvents surprisingly present comparable values of ca. −5 μV K^−1^, the PF, ultimately, presents similar trends with *σ*.

To realize the electrical conduction in SL-Ti_3_C_2_T_x_ films, that is, the electrons are transported from one MXene nanosheet to another, both the degree of stacking between MXene nanosheets and the contacts between them directly influence the charge transport behavior [59]. Therefore, the ultraviolet–visible–near-infrared (UV–vis–NIR) absorption spectra were carried out on Ti_3_C_2_T_x_ dispersions in various solvents, as shown in Fig. S6, to understand the influences of solvents on nanosheet stacking behaviors in the solution state. No obvious difference among these spectra can be found, manifesting that MXene nanosheets share similar dispersing and stacking behaviors in various solvents and further evidencing that the differences in stacking modes originate from the film-forming process. As shown in Fig. S7, different from the free-standing Ti_3_C_2_T_x_ thin film processed by DI-water, the treatment of DMF and EtOH leads to the powder-like macroscopic morphologies of MXene films on filter paper, indicating that organic solvents significantly weaken the film-forming ability of Ti_3_C_2_T_x_. FE-SEM images of organic solvents treated MXene films revealed a similar random distribution of nanosheets in both in-plane and out-of-plane directions as depicted in Fig. S8. In order to further unlock the relationship between dispersing solvents and crystallinity of MXene films, grazing incidence wide-angle X-ray scattering (GIWAXS) measurements were taken and the results are shown in Figs. 2b, S9 and S10. Both ML- and SL-Ti_3_C_2_T_x_ films obtained by using DI-water as solvent without thermal annealing exhibit good crystallinity and relatively high orientations [56], determining the interlayer spacings to be 12.55 and 13.76 Å, respectively. This indicates that the water molecules are successfully intercalated into the nanosheets of ML-MXenes upon ultrasonication, making them separated into SL-MXenes. The *d*-spacing of SL-MXene decreases to 13.20 Å via thermal annealing, and the relative intensity of the in-plane direction (azimuthal angle, χ = 0°) to the out-of-plane direction (χ = 90°) slightly increases in the meantime. This phenomenon suggests that the MXene nanosheets are more closely stacked with the removal of water molecules, yet the orientation becomes a little worse. By contrast, the MXene films obtained by dispersing in organic solvents exhibit relatively poor crystallinity and weak orientations, especially in EtOH-based suspensions. The *d*-spacing of Ti_3_C_2_T_x_ film obtained by EtOH-based suspensions without thermal annealing is determined to be 12.54 Å, nearly the same as the ML-Ti_3_C_2_T_x_ film, indicating EtOH molecules failed to massively intercalate into MXene nanosheets due to its low boiling point and hence fast evaporation during vacuum filtration. In stark contrast, DMF solvent with higher boiling point and larger size raises the interlayer spacing of MXene film to 14.88 Å. After thermal annealing at 200 °C, despite *d*-spacings drop down to 11.31 and 13.82 Å for EtOH and DMF-processed MXene films, respectively, the crystallinity and orientation of (*002*) plane are both greatly deteriorated, which is unfavorable for the acquisition of outstanding electrical conductivities.

To better understand the effects of thermal annealing on the TE performance of MXene films, thermogravimetric analysis (TGA) was conducted. It can be easily observed that SL-Ti_3_C_2_T_x_ experiences two stages of mass loss during the heating process (Fig. 2c). Given the temperature range of mass loss, it can be inferred that the reduction of mass below 200 °C is mainly caused by the removal of residual water, while the decrease above 200 °C is due to the removal of water molecules intercalated into MXene layers. By contrast, Ti_3_C_2_T_x_ films dispersed in organic solvents display mass changes in three distinct stages as shown in Fig. 2d, e. In DMF-processed MXene films, the first stage of mass loss refers to the removal of water, while the second stage represents the removal of intercalated DMF molecules. After experiencing a plateau period, the mass loss in the third stage above 400 °C may indicate the detachment of surface functional groups [49]. Similar phenomenon is also found in EtOH-processed MXene films, and the minimal mass loss implies that EtOH molecules failed to intercalate extensively into the nanosheets of MXene, in accordance with the GIWAXS results. Based on both GIWAXS and TGA results, it can be thus concluded that solvent molecules with small size and low boiling point are easier to be removed during vacuum filtration and thermal annealing processes, which aids to mitigate the destruction of residual solvents on the electrical conduction of MXene films. However, the very fast evaporation of solvent is detrimental to the realization of close stacking between MXene nanosheets.

XPS measurements were further taken to decipher the underlying in-depth mechanisms that led to the tremendous differences in the *σ* of Ti_3_C_2_T_x_ thin films with various dispersing solvents as shown in Figs. S11 and S12. The proportion of [Ti–O] to the overall Ti elements is significantly higher in MXene films obtained from DI-water-based suspensions (11.9% in ML-MXene and 14.8% in SL-MXene) than in DMF (9.2%)- and EtOH (10.8%)-based suspensions (Fig. 2f), demonstrating that the amount of surface terminal groups of −O and −OH is significantly higher in SL-MXene than in other films. Moreover, the ratio of [C–Ti–O] to [C–Ti–OH] according to O 1*s* peak as shown in Fig. 2g is greatly enhanced from 1.05 in ML-MXene film to 1.56 in SL-MXene film, revealing that −OH surface terminal groups are largely transformed into −O terminal groups in SL-MXene, which is exceptionally beneficial to the elevation of *σ* [51]. Thus, the superior *σ* in SL-Ti_3_C_2_T_x_ film results mainly from the following two aspects: (1) the tight stacking between MXene nanosheets and the high orientation of (*002*) plane in parallel to the substrate; (2) the surface terminal groups of −OH and −F are largely substituted by −O.

### Nanosheet Stacking of MXene Films

**Fig. 3:**
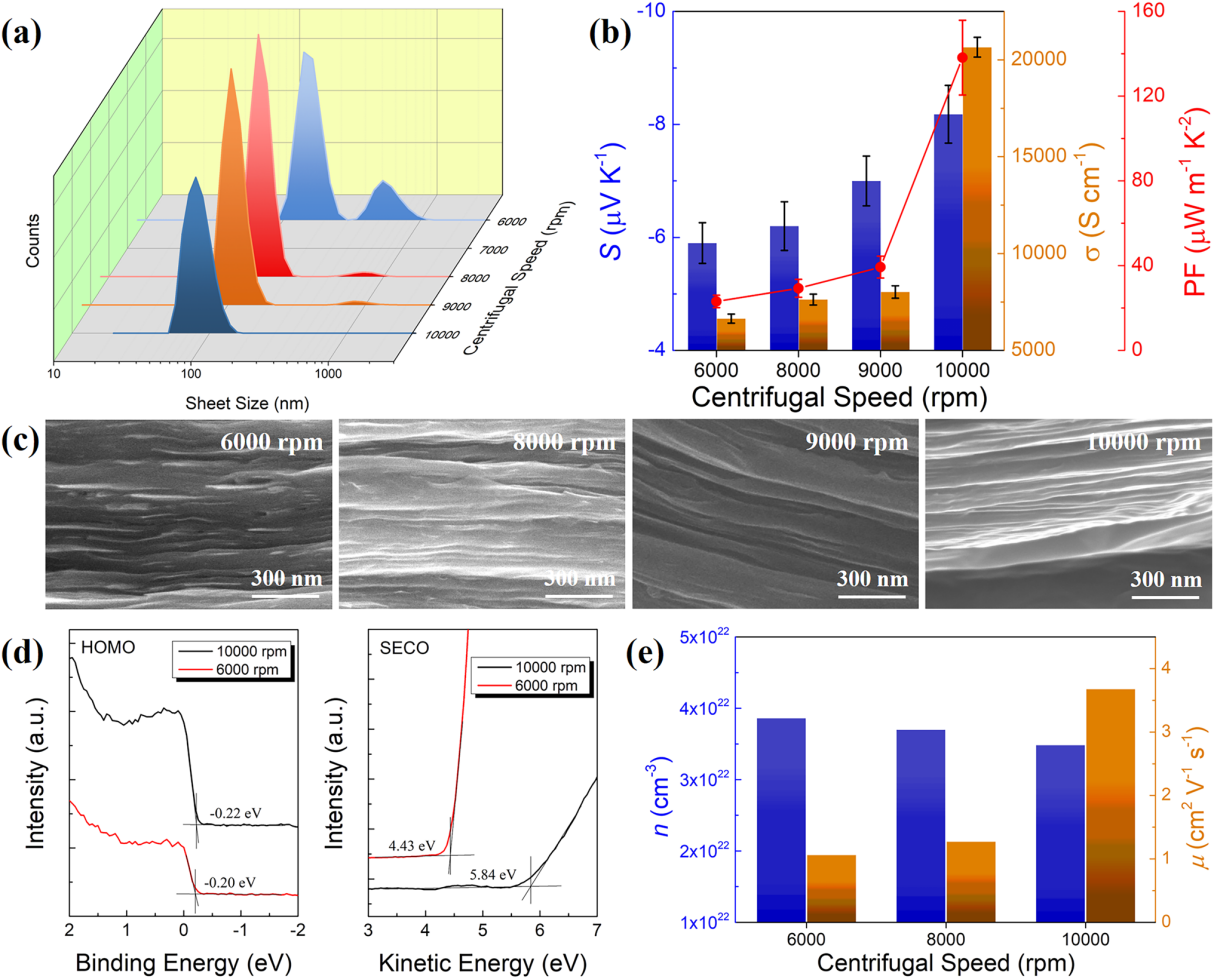
Effects of nanosheet size on Ti_3_C_2_T_x_ MXene films. **a** Distribution of nanosheet sizes of Ti_3_C_2_T_x_ suspensions obtained at different centrifugal speeds. **b** TE performance of *σ*, *S* and PF of single-layered Ti_3_C_2_T_x_ thin films processed at various centrifugal speeds. **c** Cross-sectional SEM images of Ti_3_C_2_T_x_ thin films with various nanosheet sizes. **d** UPS profiles of Ti_3_C_2_T_x_ thin films processed at a centrifugal speed of 10,000 and 6000 rpm, respectively. **e** Determination of carrier concentration and Hall mobility of Ti_3_C_2_T_x_ thin films.

The above study has now determined that DI-water is more favorable to the TE performance of Ti_3_C_2_T_x_ MXene films, and hence the following research will focus on DI-water as the dispersing solvent. As a class of two-dimensional materials, the size of MXene nanosheets has significant effects on their TE properties [27]. In order to unveil their relationship, the Ti_3_C_2_T_x_ films with different sheet size distributions were achieved by applying high-speed centrifugation at a rate of 10,000, 9,000, 8,000 and 6,000 rpm, respectively. It has to be emphasized that the formation of small-sized MXene nanosheets was the result of a long-duration and high-intensity ultrasonication process, which tore large-sized MXene sheets into smaller ones. The distribution of nanosheet sizes in MXene suspensions processed at different centrifugal speeds is shown in Fig. 3a. The corresponding average nanosheet sizes are estimated to be 80, 106, 122 and 142 nm at centrifugal speeds of 10,000, 9,000, 8,000 and 6,000 rpm, respectively. Among them, the MXene suspensions centrifuged at 6,000 rpm exhibit the most evident bimodal distribution with another average nanosheet size of 531 nm. In addition, the distribution of nanosheet sizes in the solid state was observed by TP-AFM tests, more accurately reflecting the state of nanosheets in filtered thin films. As shown in Fig. S14, only the MXene film obtained by centrifugation at 6000 rpm contains both relatively larger SL-MXene nanosheets (average size of 600–800 nm) and smaller ones (average size of 100–200 nm), indicating the existence of bimodal distribution of nanosheet sizes. By contrast, the film centrifuged at 10,000 rpm displays the most uniform distribution of MXene nanosheet sizes with virtually all smaller than 100 nm. The reason for this phenomenon is that high-speed centrifugation would lead to the precipitation of large-sized nanosheets, yet small-sized ones can be retained in the supernatant and hence MXene film with a distribution of smaller-sized MXene nanosheets can be realized by higher centrifugal speed. It is observed that the size distribution of nanosheets obtained at 10,000 rpm centrifugation is already very narrow, and thus a higher centrifugal speed is difficult to yield better separation of MXene nanosheets, thereby determining 10,000 rpm to be the highest centrifugal speed utilized in this study.

The *S* and *σ* of MXene thin films centrifuged at various speeds were measured as shown in Fig. 3b. With the elevation of centrifugal speed, the *σ* shows a monotonically increasing trend from 6639 S cm^−1^ at 6000 rpm to 20,652 S cm^−1^ at 10,000 rpm, the latter of which is also among the best in the literature [16, 17]. Cross-sectional SEM images (Fig. 3c) present that MXene films obtained by centrifugal speed no more than 9,000 rpm possess many wrinkles and voids in the cross-sectional regions, indicative of relatively poor nanosheet stackings. In comparison, the microstructures of the 10,000 rpm processed MXene film at the cross-sectional region are more uniform with fewer wrinkles and better stacking density. This is presumably due to the fact that the smaller-sized nanosheets can effectively fill the gaps between MXene layers, thus enhancing the contacts between them and aiding in the effective conductive pathway. Of particular note, the time of vacuum filtration process is also significantly prolonged with the increase of centrifugal speed of MXene suspensions, which confirms the ultra-close stacking of MXene nanosheets during the filtration process. Moreover, the *S* of MXene films underwent a similar trend with *σ* upon increasing centrifugal speed, as shown in Fig. 3b. The highest *S* of −8.2 μV K^−1^ was realized in 10,000 rpm centrifuged MXene film, exhibiting a ~ 39% enhancement relative to the *S* of − 5.9 μV K^−1^ in 6,000 rpm centrifuged film. The simultaneous improvement in both *S* and *σ* via an uplift of centrifugal speed resulted in the surprisingly reinforced PF from 23.1 ± 2.9 to 138.2 ± 17.7 μW m^−1^ K^−2^, which is, to our knowledge, the highest value reported for neat MXene films at room temperature as shown in Table S1 [49–51]. Obviously, the variation in stacking morphologies of MXene nanosheets solely cannot explain the increase in *S* and the decoupling of *S* and *σ*. To gain more insights into the underlying mechanisms, ultraviolet photoelectron spectroscopy (UPS) was used to monitor the energy-level structures of MXene films as shown in Fig. 3d. The Fermi level (*E*_F_) of Ti_3_C_2_T_x_ film is significantly downward shifted from −4.43 to −5.84 eV by increasing the centrifugal speed from 6000 to 10,000 rpm. The deepening of *E*_F_ is coincided with the phenomenon that surface terminal groups change from −OH and −F to −O [60], which would also lead to the boost in *σ* of MXene films. The carrier concentration (*n*) and carrier mobility (*μ*) of MXene films were further determined by Hall measurements as shown in Fig. 3e. The *n* drops very slightly with the increase of centrifugal speed, which are all in the range of 3.4–3.9 × 10^22^ cm^−3^, consistent with the literature values [51]. On the contrary, the *μ* is greatly enhanced with the increase of centrifugal speed and the corresponding decrease of nanosheet sizes, exhibiting the highest value of 3.68 cm^2^ V^−1^ s^−1^, which is almost 3.5 × of the MXene film processed at 6,000 rpm. It should be noted that as the average size of MXene nanosheets decreases, the same mass or volume of film would inevitably contain more nanosheets, resulting in a significant increase in the number of grain boundaries, which has a negative effect on the *μ*. However, the continuous improvement of *μ* with an increasing centrifugal speed proves that the significantly denser stacking of MXene nanosheets as evidenced in Fig. 3c exerts a greater impact on the *μ* than the higher boundary density and defects. As a result, the distinct improvement in *σ* is mainly attributed to the *μ* reinforcement, as realized by the amelioration of close stackings between MXene nanosheets. The above-mentioned substantial downshift in *E*_F_ demonstrates that the comparatively small changes in nanosheet size can cause significant changes in surface terminal groups and energy levels. The size of nanosheets in MXene thin films is not a fixed value and instead displays a certain distribution. The smaller the size of MXene nanosheets, the more interfaces between them would be generated, leading to increased scattering and large energy barriers at interfaces. Thus, high-energy electrons are allowed to pass through the interfaces, while low-energy electrons are filtered out, hence increasing the average energy of charge carriers, which is known as the energy filtering effect [61, 62], giving rise to the enhancement in *S*. Finally, a planar and flexible 5-leg thermoelectric device was constructed with the best-performing Ti_3_C_2_T_x_ thin film as the n-type leg (16.0 × 7.0 × 0.005 mm^3^). As presented in Fig. S15, the output voltage of the TE device gradually increases from 0.48 to 1.21 mV as the temperature gradient (ΔT) rises from 12 to 30 K. The average *S* of Ti_3_C_2_T_x_ film was estimated to be −8.02 μV K^−1^, which agrees very well with the measured value. The maximum output power reaches 39.64 nW at ΔT = 30 K, leaving much room for further improvement since the overall contact resistance of 9.9 Ω accounts for the majority of the internal resistance (11.0 Ω) of the device. Nonetheless, the output performance of this TE device remains comparable to those reported TE devices as shown in Table S2.

### MXene Nanocomposites

**Fig. 4:**
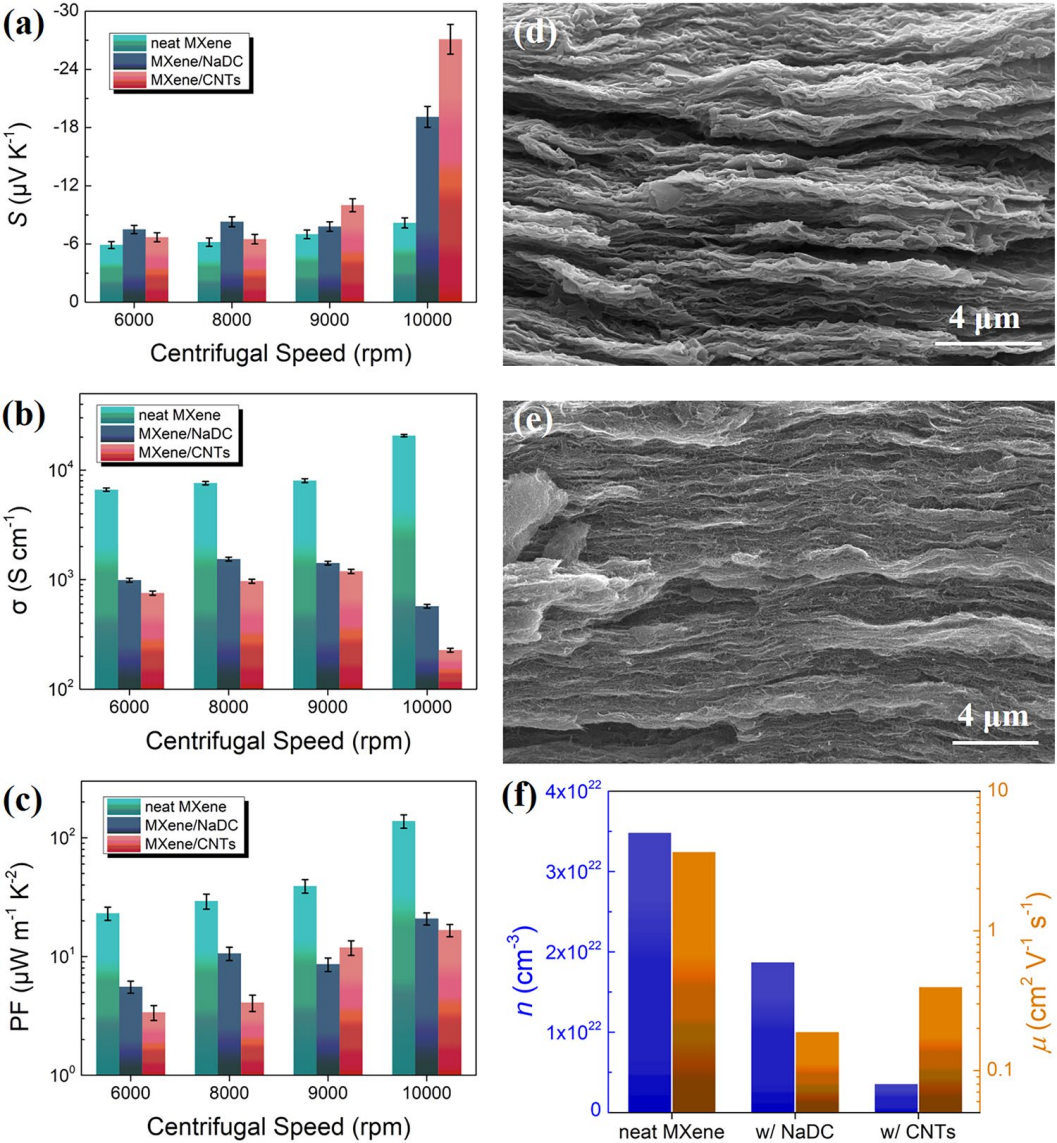
Thermoelectric properties of **a** Seebeck coefficient (*S*), **b** electrical conductivity (*σ*) and **c** power factor (PF) of single-layered Ti_3_C_2_T_x_ thin films obtained at different centrifugal speeds and its composites with NaDC and SWCNTs. Cross-sectional SEM images of **d** Ti_3_C_2_T_x_/NaDC and **e** Ti_3_C_2_T_x_/SWCNTs composites. **f** Determination of carrier concentration and Hall mobility of neat Ti_3_C_2_T_x_ thin film centrifuged at 10,000 rpm and its composite films with NaDC and SWCNTs, respectively.

We have now shown that neat Ti_3_C_2_T_x_ films exhibited excellent *σ* yet lagged in *S* far behind other n-type semiconducting materials. We were therefore tempted to fabricate MXene-based nanocomposites to enhance the *S* and investigate the corresponding PF values. Herein, SWCNTs and the amphoteric surfactant NaDC were added into Ti_3_C_2_T_x_/DI-water suspensions, followed by vacuum filtration yielding the nanocomposite films. The aim of introducing NaDC is to further ameliorate the dispersion of MXene in DI-water. Given that this work is consistently focused on MXene and the TE performance is mainly determined by the MXene characteristics, the amount of the second component was controlled at a very low level. Within the second component content range between 0.5 and 1.5 wt%, the addition of 1 wt% SWCNT and NaDC both achieved the best TE performance as shown in Fig. S16. As presented in Fig. 4a, the *S* of nanocomposite films is generally higher than that of neat MXene films, which is mostly significant in the samples processed at the highest centrifugal speed. Ultimately, a champion *S* as high as −27.1 μV K^−1^ was acquired when SWCNTs were composited with MXene centrifuged at 10,000 rpm. In the composite films of SWCNTs and Ti_3_C_2_T_x_ centrifuged at a speed below 10,000 rpm, the increase in *S* is not very evident, which may be due to the small difference in *E*_F_ between them and hence a low-energy barrier at the interfaces. By contrast, the *σ* of these nanocomposite films exhibits the completely opposite trends with MXene processed from different centrifugal speeds as shown in Fig. 4b. The *σ* of nanocomposite film is markedly reduced relative to neat MXene films and, surprisingly, displays the lowest value in MXene/SWCNTs composite film (227 S cm^−1^) with the smallest MXene sheet size although the corresponding MXene thin film possesses the highest *σ* of ~ 20,000 S cm^−1^. This phenomenon indicates that the contacts between MXene nanosheets are greatly damaged with NaDC or SWCNTs added. Finally, although both NaDC and CNTs blended with MXene centrifuged at 10,000 rpm exhibit the lowest *σ*, the significant increase in *S* aids to the elevation in PF compared to other composites as shown in Fig. 4c. Nonetheless, the over 3× higher *S* of CNTs/MXene (10,000 rpm) composite film in comparison with that of neat MXene cannot counteract the sharp decrease of *σ*, which eventually yields the remarkable drop in PF.

The microstructures of nanocomposites composed of 10,000 rpm centrifuged Ti_3_C_2_T_x_ with NaDC or SWCNTs were further characterized to understand the effects of constructing nanocomposites on TE performance. Since NaDC cannot be clearly observed in SEM image as shown in Fig. 4d, the EDS elemental mapping was carried out and Na element was evenly distributed similar to C, O and Ti, as shown in Fig. S17, indicating the successful insertion of NaDC molecules into MXene nanosheets. A large number of wrinkles and voids emerge in the nanocomposite films (Figs. 4d, e and S18), especially in MXene/NaDC composite films. Thus, the original tightly stacked morphologies of MXene films were damaged by the introduction of NaDC and CNTs, which should be responsible for the substantial reduction in *σ*. Given the high carrier mobility in CNTs [63], the inserted CNTs may act as part of the conductive paths, assuring the *σ* not to be greatly impaired. These results explicitly suggest that the stacking of MXene nanosheets is sensitive to the introduction of other components. The XRD measurements as depicted in Fig. S19 witness a strange phenomenon that the intensity of (*002*) peak is significantly enhanced relative to SL-MXene film and several peaks including (*004*), (*006*), (*101*), (*103*), (*104*), (*105*) and (*106*), which are invisible in neat MXene films reoccurred in nanocomposites. It can be explained as the introduction of SWCNTs and NaDC causes the aggregation of MXene, which significantly enhances its crystallinity. Yet, the aggregated MXene nanosheets are severely separated by SWCNTs or NaDC, leading to the poor overall microstructures. Additionally, the (*002*) peak shifts to lower degrees in both nanocomposites, suggesting that SWCNTs and NaDC are slightly intercalated into MXene layers.

Hall measurements were exploited to determine the carrier concentration and mobility of the above nanocomposites to elucidate the varied TE performance as shown in Fig. 4f. The *n* of MXene composites with NaDC and SWCNTs both greatly decrease relative to neat MXene films processed at a centrifugal speed of 10,000 rpm (3.5 × 10^22^ cm^−3^), while the former (1.9 × 10^22^ cm^−3^) almost halves and the latter (3.6 × 10^21^ cm^−3^) even reduces by an order of magnitude. The huge reduction of *n* in CNTs-based composites is mainly owing to the introduction of p-type CNTs into the n-type matrix of MXene and the consequent counteractions between holes and electrons, resulting in the decrease of electron concentration [28, 30]. The transfer of electrons from Ti_3_C_2_T_x_ MXene to SWCNTs and NaDC can be evidenced by the XPS results as shown in Figs. S20-S22. In the meantime, the decrease of *n* gives rise to the elevation of *S*, which is also a result of energy filtering effect due to the rich interfaces between MXene nanosheets and NaDC or CNTs as depicted in Fig. 4d, e. The *μ* of MXene/NaDC composites (0.19 cm^2^ V^−1^ s^−1^) is equivalent to 5.1% of 10,000 rpm centrifuged MXene film, evidencing that the voids and wrinkles in nanocomposite films significantly hinder the electron transportation. The more than doubled *μ* of MXene/SWCNTs composites as high as 0.40 cm^2^ V^−1^ s^−1^ indicates that CNTs can serve as a supplement to conductive pathways, effectively alleviating the significant decrease in *μ* caused by poor morphologies and loose nanosheet stacking. Moreover, the simultaneous decrease of both *n* and *μ* with the introduction of NaDC and CNTs is responsible for the significant lowering of *σ*. Therefore, the addition of NaDC and CNTs disrupts the continuity between MXene nanosheets, which significantly undermines *σ* to the extent that even several-folds increase in *S* cannot counteract it. Finally, the temperature-dependent TE performance was measured (Fig. S23), and the optimal PF reaches 37.6 μW m^−1^ K^−2^ at 348 K for 1 wt% SWCNT/Ti_3_C_2_T_x_ film and 64.8 μW m^−1^ K^−2^ at 369 K for 1 wt% NaDC/Ti_3_C_2_T_x_ film, respectively.

## Conclusions

In closing, we have for the first time conducted a systematic investigation on thermoelectric modulation of neat Ti_3_C_2_T_x_ MXene films. The study began with an exploration of various dispersing solvents for Ti_3_C_2_T_x_ thin films and discovered that deionized water was superior for achieving closely packed single-layered MXene nanosheets. This was conducive to obtaining more than one order of magnitude higher *σ* than other polar solvents of DMF and EtOH. Subsequently, the impact of the centrifugal speed on SL-Ti_3_C_2_T_x_ suspensions was examined, unveiling that higher speeds were beneficial in obtaining smaller-sized MXene nanosheets, which promoted extremely tight stacking and high orientation of MXene nanosheets, resulting in an extremely high *σ* of ~ 20,000 S cm^−1^. Meanwhile, the *S* also increased with higher centrifugal speeds, which is attributed to the energy filtering effect between MXene nanosheets with diverse energy landscapes and surface terminal groups, leading to an ultrahigh power factor up to ~ 156 μW m^−1^ K^−2^ at room temperature. Furthermore, nanocomposites incorporating MXene with tiny amounts of NaDC or SWCNTs were fabricated to greatly raise *S* yet reduce *σ*. Our study deciphers the key factor of nanosheet stacking of Ti_3_C_2_T_x_ thin films on the thermoelectric performance, which may pave the way for and accelerate future exploration of this highly awaiting field in MXenes.

## Supplementary Information

Below is the link to the electronic supplementary material.

Supplementary file1 (DOCX 8884 KB)
