# Supplementary material for: Thermoelectric Modulation of Neat Ti3C2Tx MXenes by Finely Regulating the Stacking of Nanosheets
Source: Nanomicro Lett. 2024 Dec 26;17:93. doi: 10.1007/s40820-024-01594-z (PMC11671675; doi:10.1007/s40820-024-01594-z)
Supplement: Supplementary file 1 — Supplementary file1 (DOCX 8884 KB) [file 40820_2024_1594_MOESM1_ESM.docx]

Supporting Information for

**Thermoelectric Modulation of Neat Ti_3_C_2_T_x_ MXenes by Finely Regulating the Stacking of Nanosheets**

Junhui Tang^1,⊥^, Renyang Zhu^1,⊥^, Ya-Hsin Pai^1,⊥^, Yan Zhao^1,^ *, Chen Xu^2,^ * and Ziqi Liang^1,^ *

^1^ Department of Materials Science, Fudan University, Shanghai, 200433, P. R. China

^2^ Spine Center, Department of Orthopedics, Shanghai Changzheng Hospital, Naval Medical University, Shanghai, 200003, P. R. China

^⊥^ Junhui Tang, Renyang Zhu and Ya-Hsin Pai contributed equally to this work.

*Corresponding authors. E-mail: [zqliang@fudan.edu.cn](mailto:zqliang@fudan.edu.cn) (Ziqi Liang); [zhaoy@fudan.edu.cn](mailto:zhaoy@fudan.edu.cn) (Yan Zhao); [chenxu1988@smmu.edu.cn](mailto:chenxu1988@smmu.edu.cn) (Chen Xu)

**Supplementary Figures**


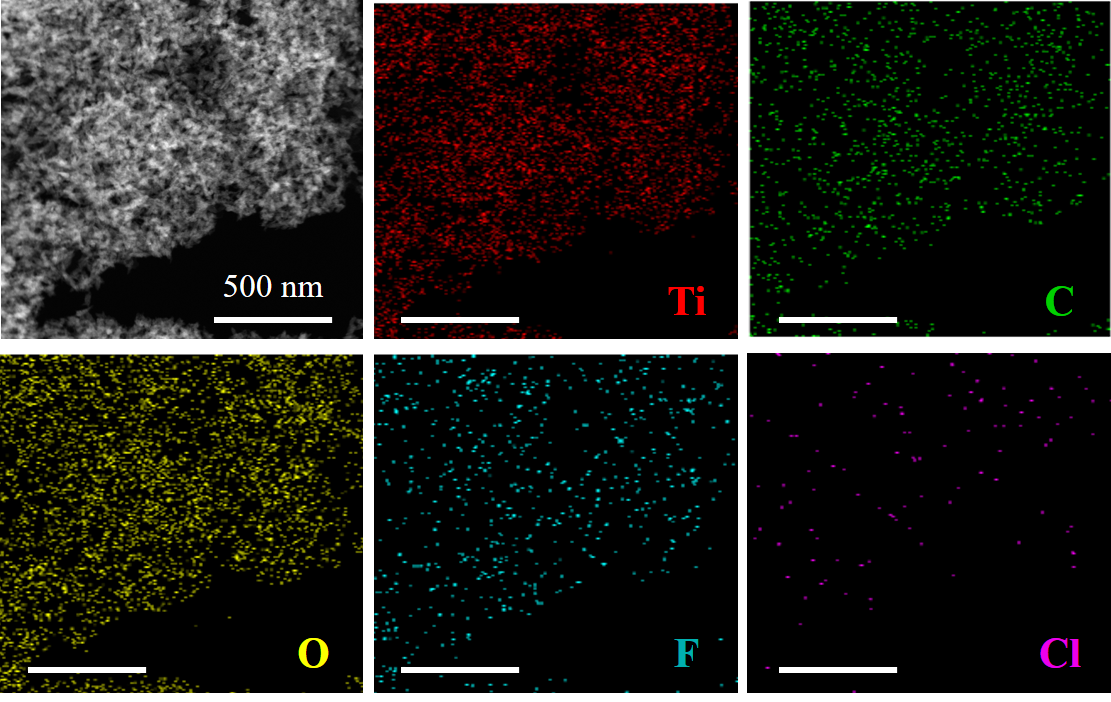


**Fig. S1** Low-magnification TEM image of SL-Ti_3_C_2_T_x_ and corresponding energy dispersive X-ray spectroscopy (EDS) elemental mapping images of Ti, C, O, F and Cl. The scale bar is 500 nm in all images


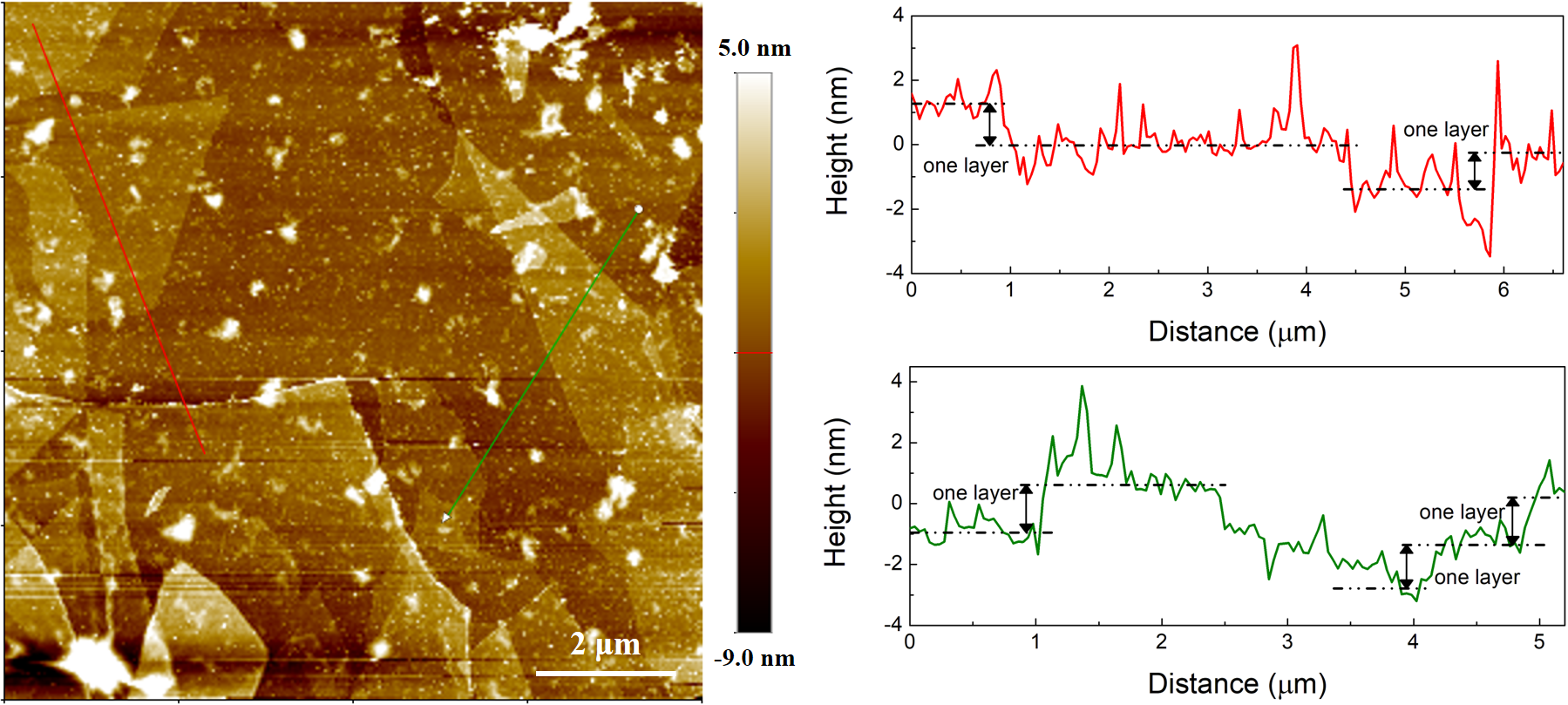


**Fig. S2** TP-AFM image of spin-coated SL-Ti_3_C_2_T_x_ thin film and the corresponding height profiles of red and green lines in the AFM image


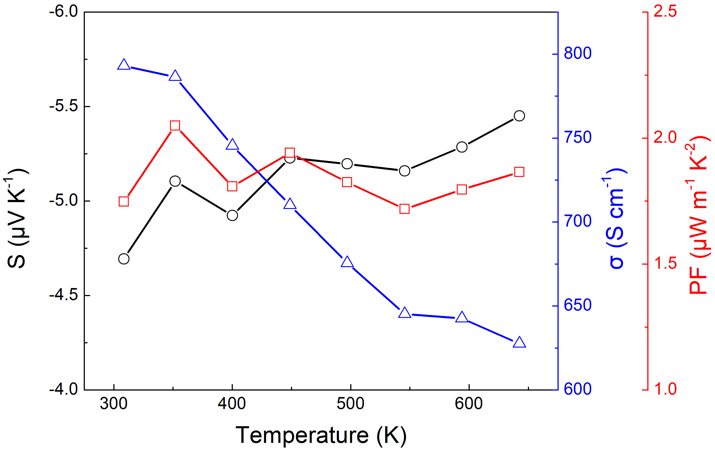


**Fig. S3** Temperature-dependent thermoelectric properties of ML-Ti_3_C_2_T_x_ thin film during the cooling process


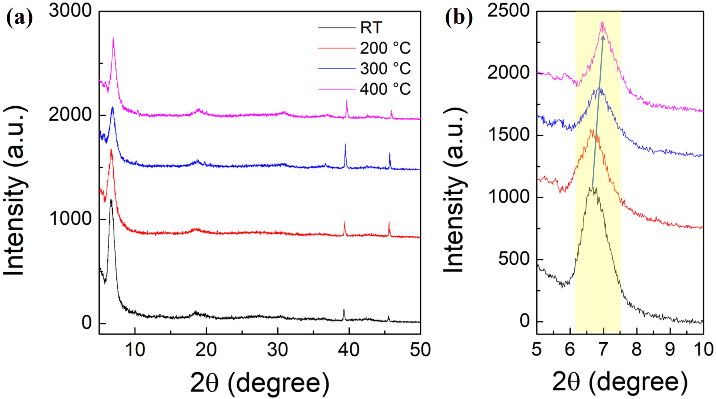


**Fig. S4** XRD patterns of the as vacuum filtered SL-MXene film and films that are thermally annealed at various temperatures


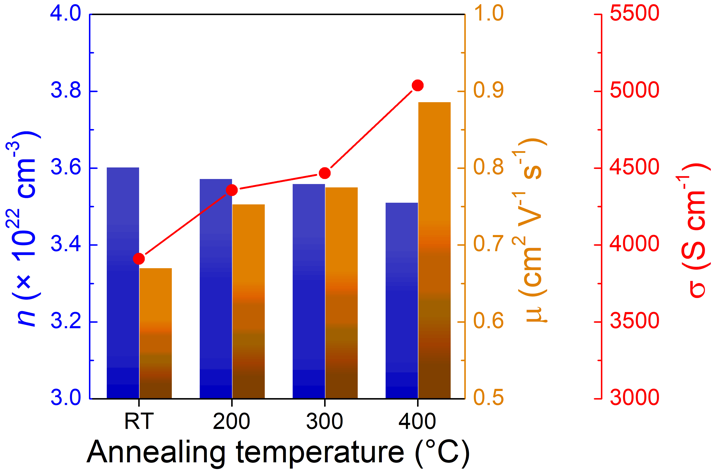


**Fig. S5** The carrier concentration and Hall mobility of Ti_3_C_2_T_x_ thin films that are thermally annealed at various temperatures


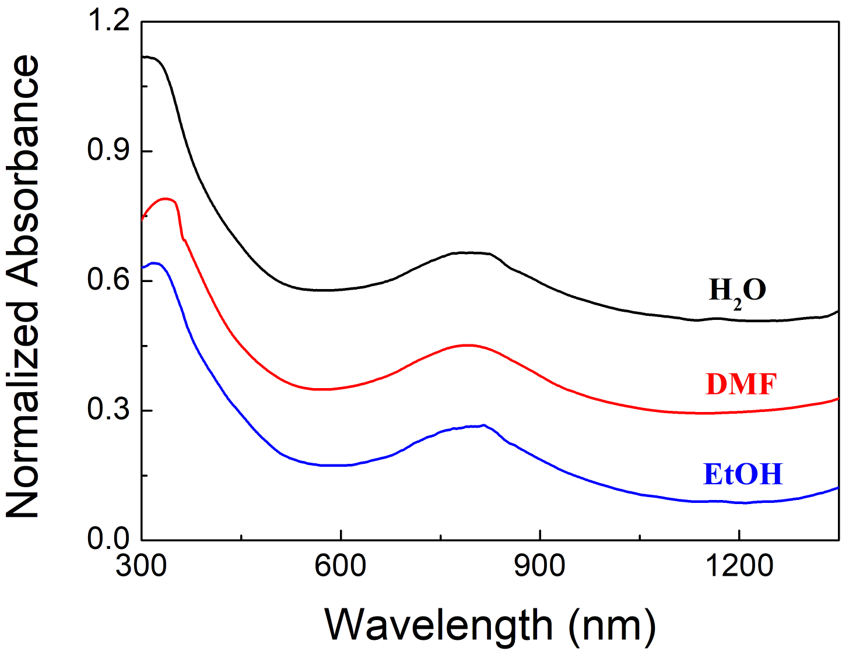


**Fig. S6** The normalized UV−vis−NIR absorption spectra of Ti_3_C_2_T_x_ MXene suspensions with DI-water, DMF and EtOH as dispersing solvents


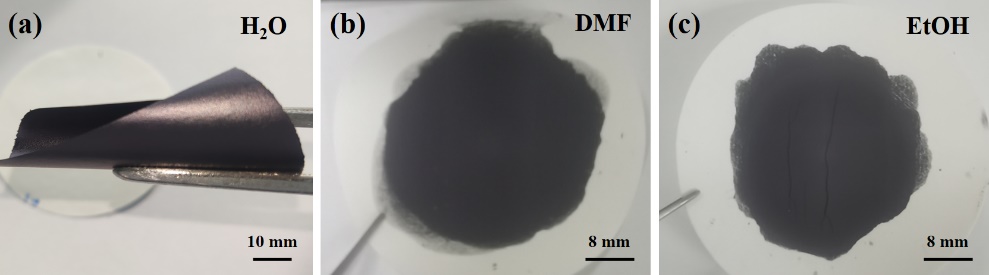


**Fig. S7** Photographs of Ti_3_C_2_T_x_ films obtained by dispersions in solvents of (**a**) DI-water, (**b**) DMF and (**c**) EtOH, respectively


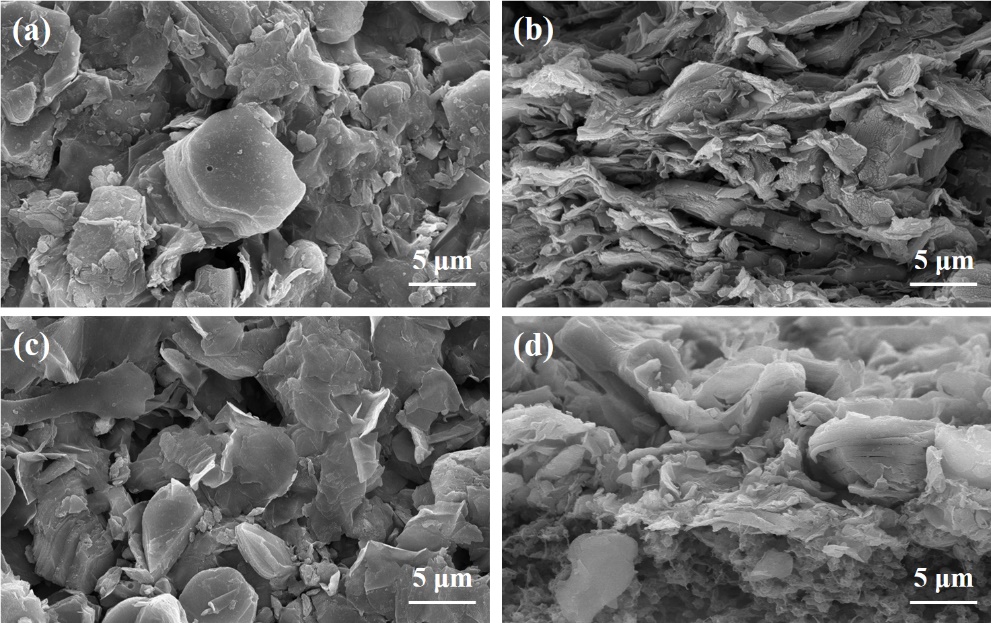


**Fig. S8** FE-SEM images of the (**a, c**) surface and (**b, d**) cross-sectional morphologies of (**a, b**) DMF and (**c, d**) EtOH processed Ti_3_C_2_T_x_ MXene films


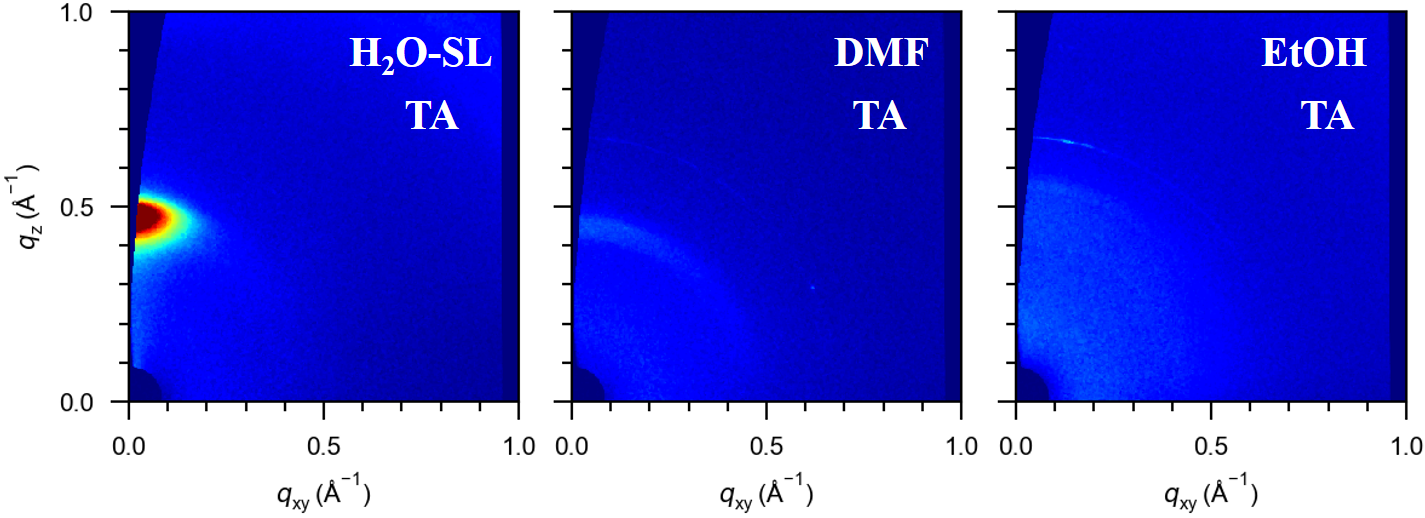


**Fig. S9** GIWAXS patterns of SL-Ti_3_C_2_T_x_ thin film acquired by H_2_O-based suspensions and Ti_3_C_2_T_x_ thin films obtained by DMF- and EtOH-based suspensions after thermal annealing at 200 ℃, respectively


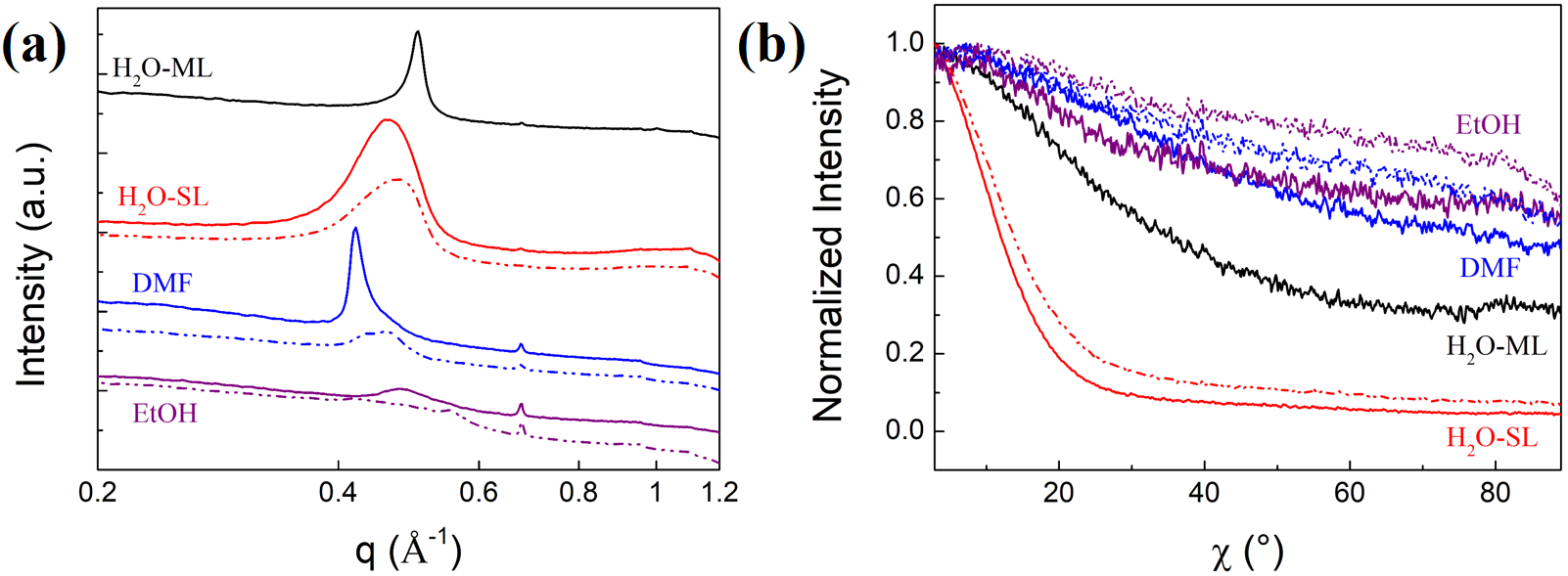


**Fig. S10** The integration curves as a function of (**a**) q and (**b**) χ derived from GIWAXS profiles of ML- and SL-Ti_3_C_2_T_x_ thin film acquired by H_2_O-based suspensions as well as Ti_3_C_2_T_x_ thin films obtained by DMF- and EtOH-based suspensions. The solid and dash-dot lines refer to MXene films before and after thermal annealing at 200 ℃, respectively


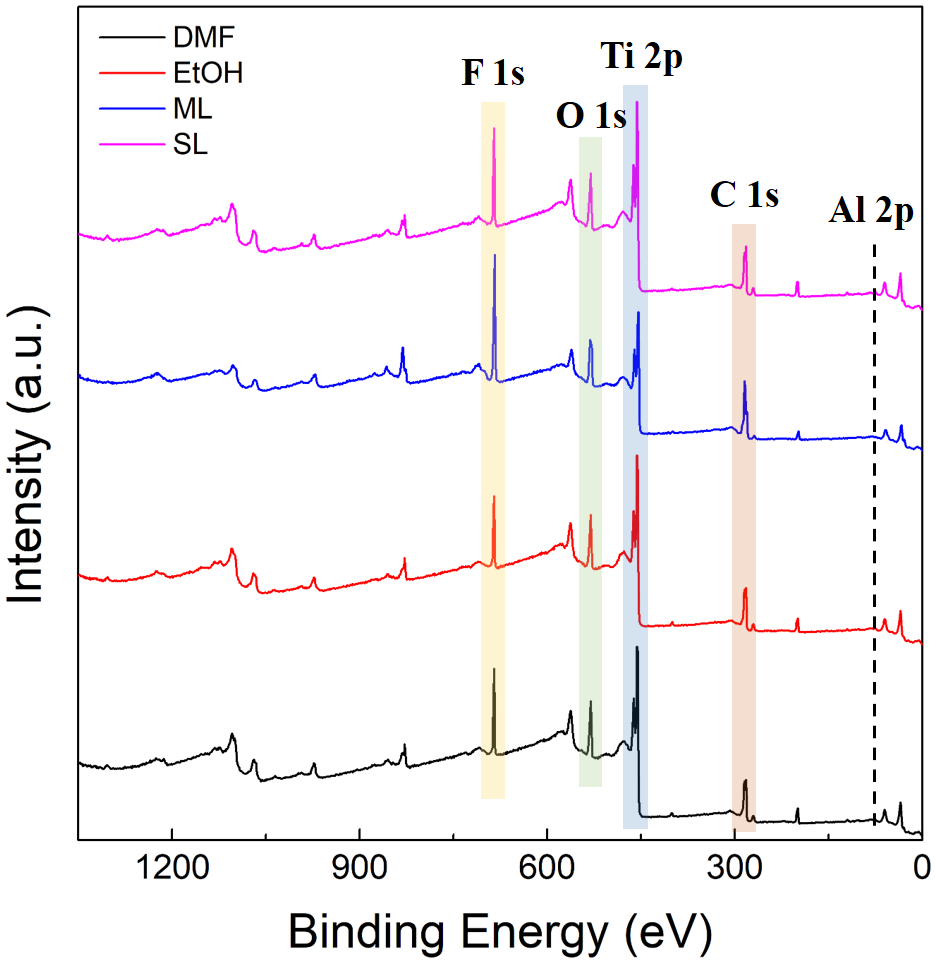


**Fig. S11** XPS survey scan of Ti_3_C_2_T_x_ thin films processed with various dispersing solvents


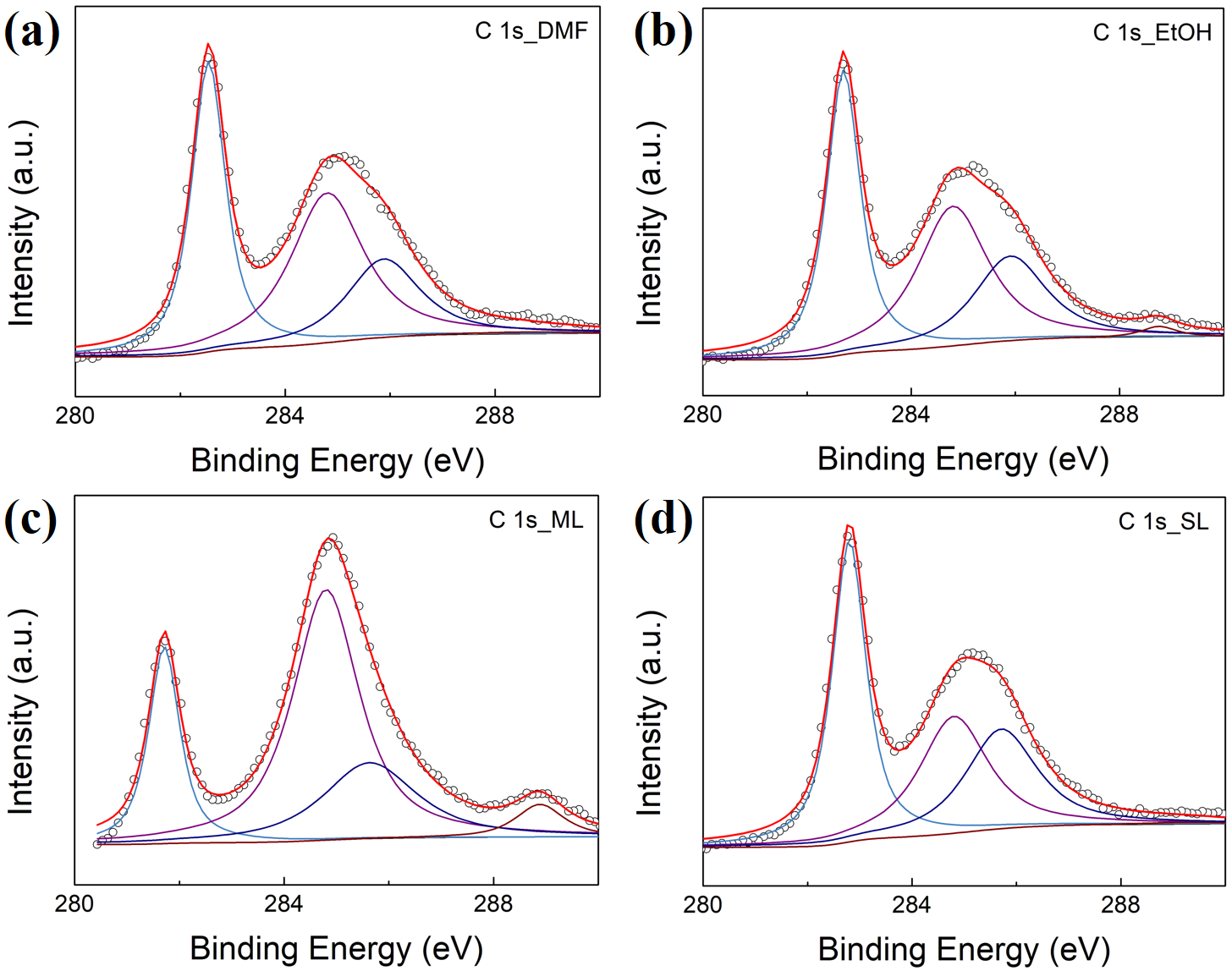


**Fig. S12** XPS patterns of C 1*s* for Ti_3_C_2_T_x_ thin films processed with various dispersing solvents


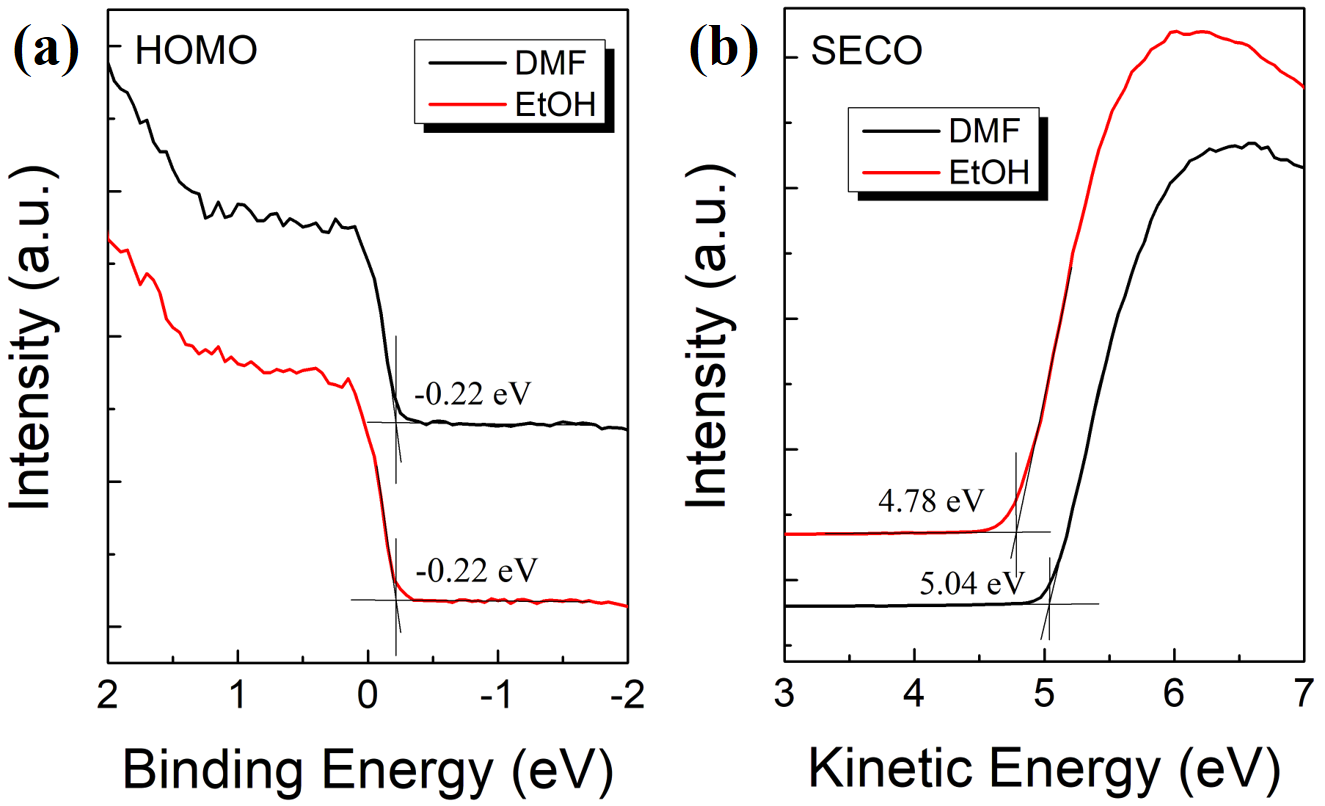


**Fig. S13** UPS results of Ti_3_C_2_T_x_ thin films processed by DMF and EtOH solvents, respectively


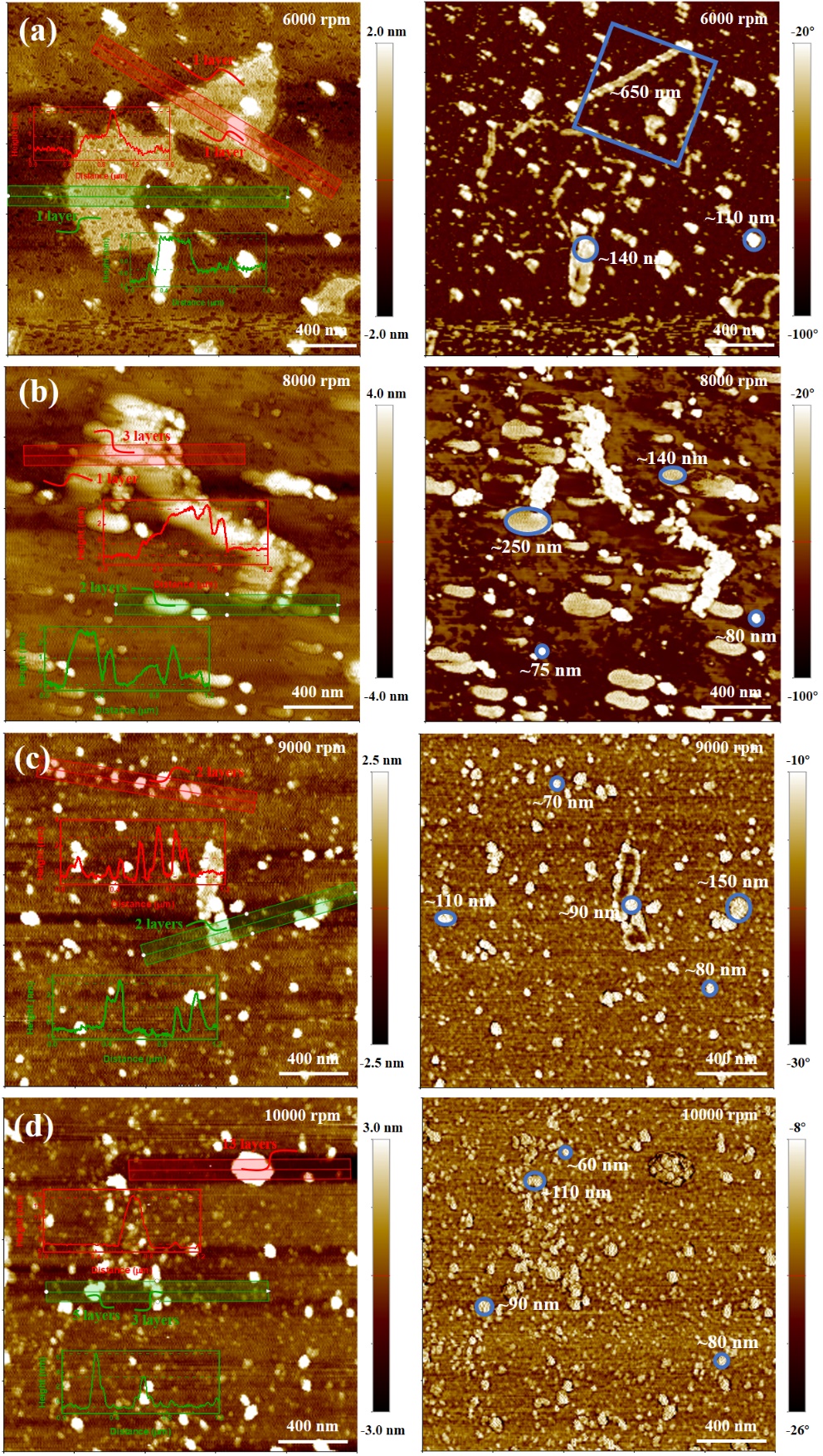


**Fig. S14** TP-AFM images of spin-coated Ti_3_C_2_T_x_ thin films via MXene dispersions centrifuged at (**a**) 6000, (**b**) 8000, (**c**) 9000 and (**d**) 10000 rpm, respectively


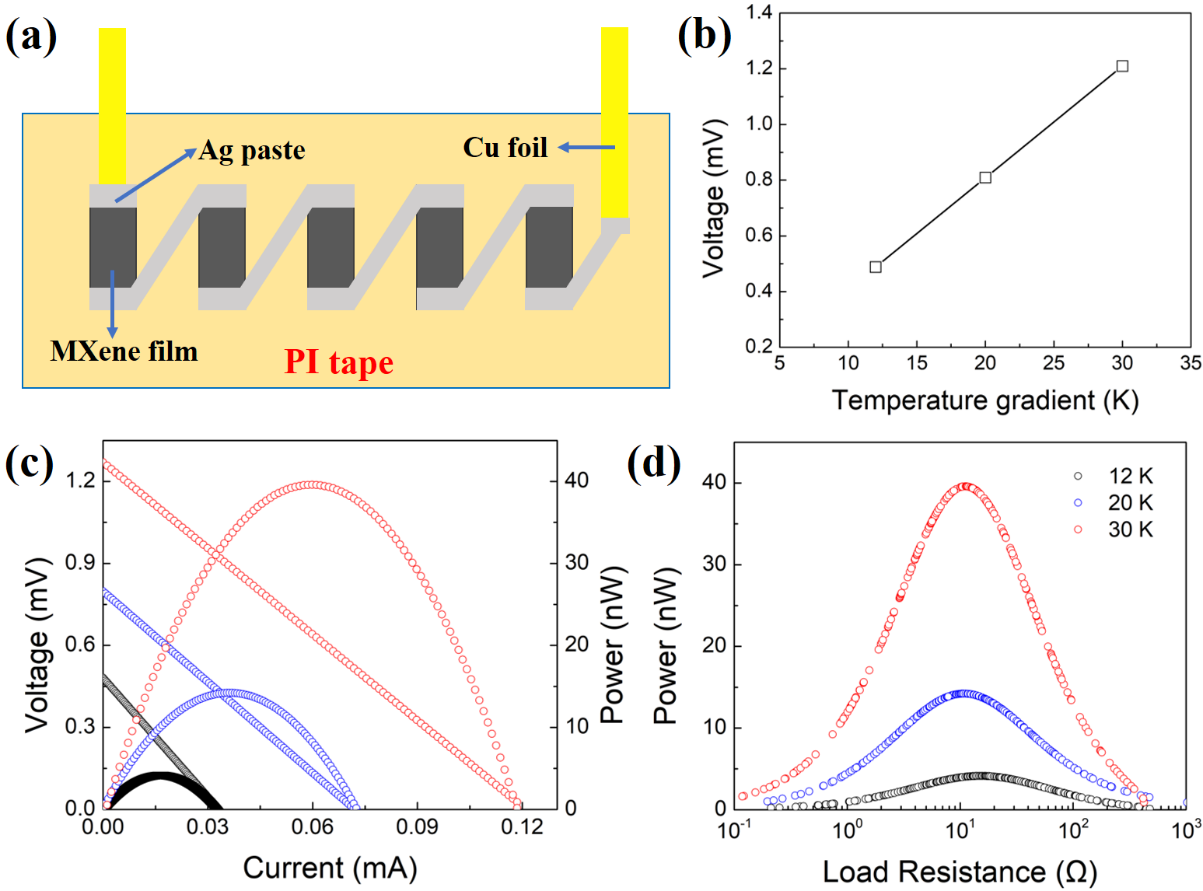


**Fig. S15** (**a**) Schematic of the flexible planar TE device consisting of 5 n-legs. (**b**) Open-circuit voltage as a function of temperature gradient. (**c**)The voltage−current and power−current curves of the TE device at different temperature gradients. (**d**) The dependence of output power on load resistance


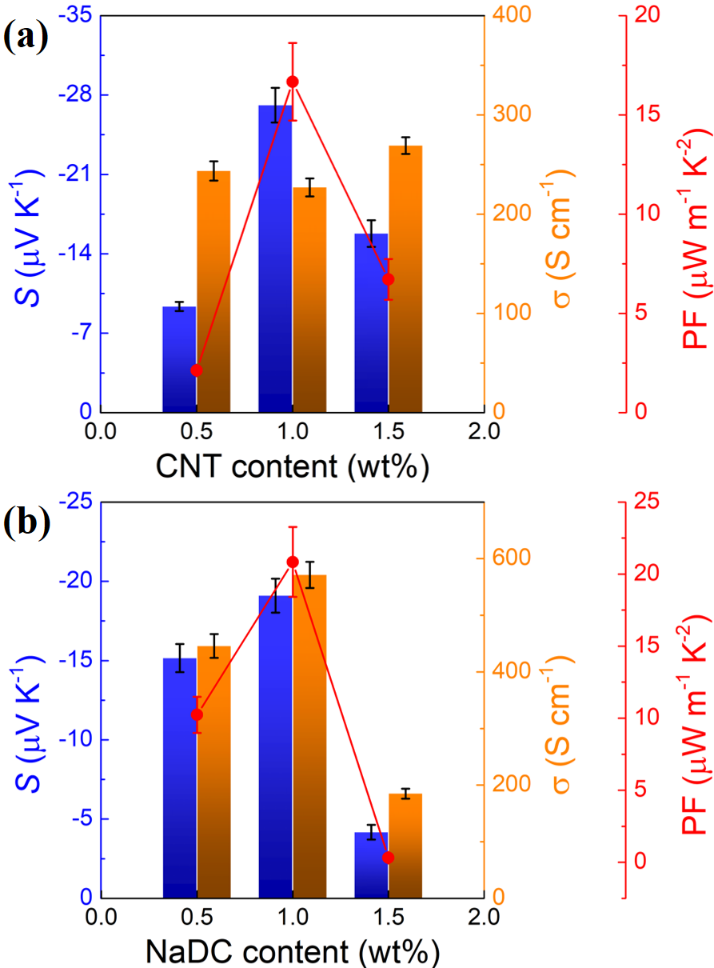


**Fig. S16** TE performance of nanocomposites consisting of Ti_3_C_2_T_x_ MXene processed at 10000 rpm centrifugation with various (**a**) SWCNT and (**b**) NaDC contents


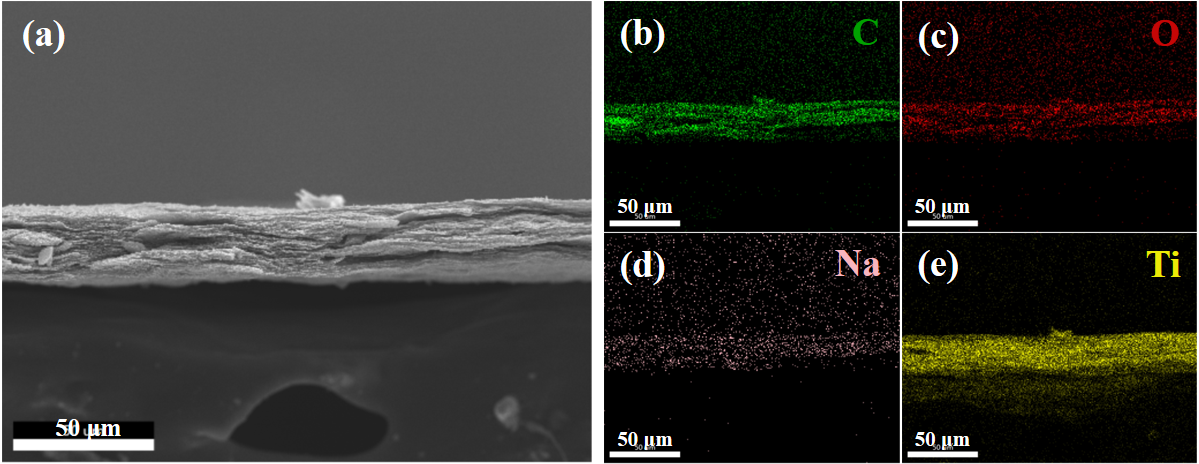


**Fig. S17** (**a**) The cross-sectional SEM image of Ti_3_C_2_T_x_/NaDC composite film and corresponding (**b−e**) elemental (−C, −O, −Na, and −Ti) mapping


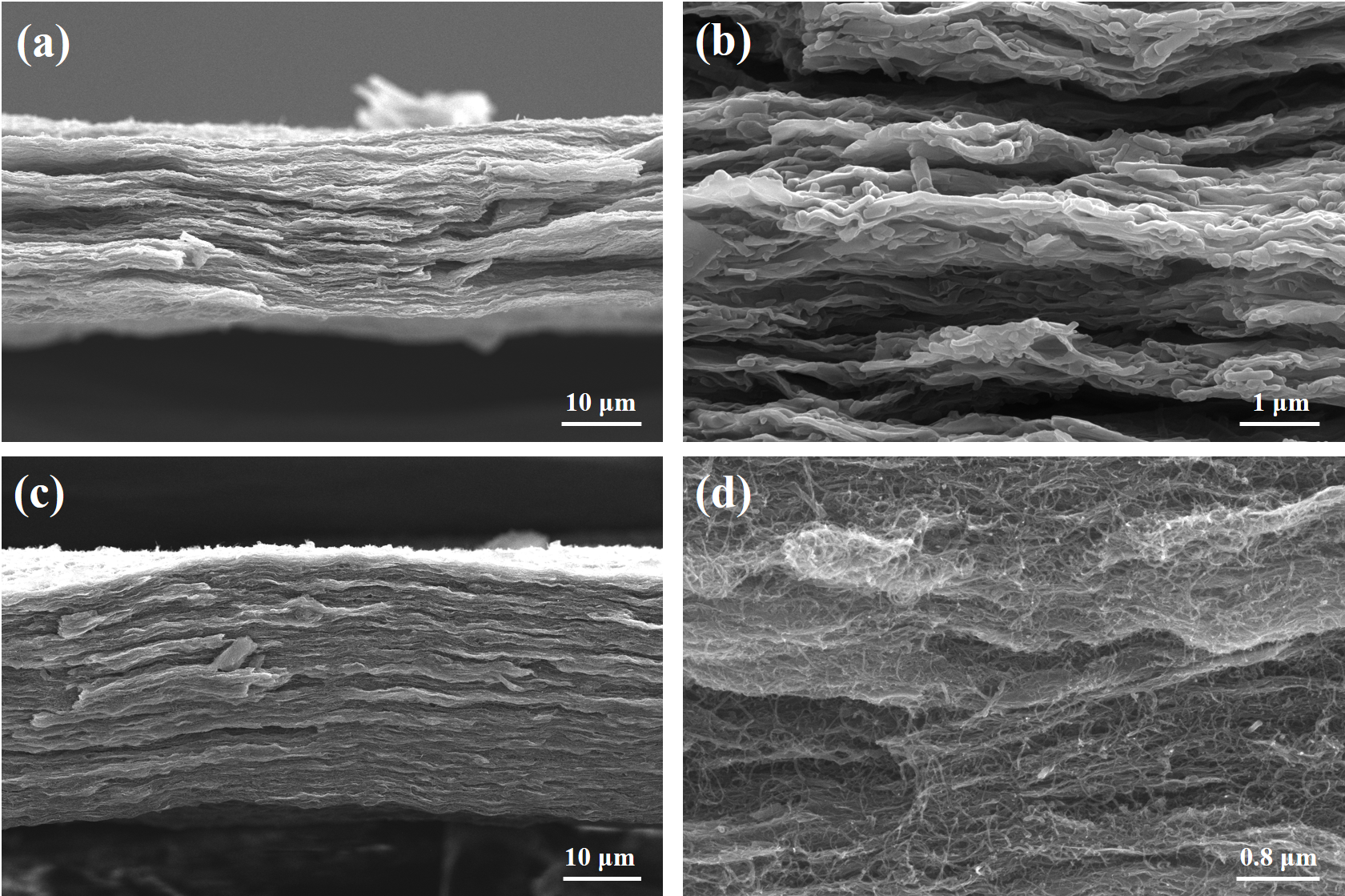


**Fig. S18** Cross-sectional SEM images of (**a, b**) Ti_3_C_2_T_x_/NaDC and (**c, d**) Ti_3_C_2_T_x_/CNT composites


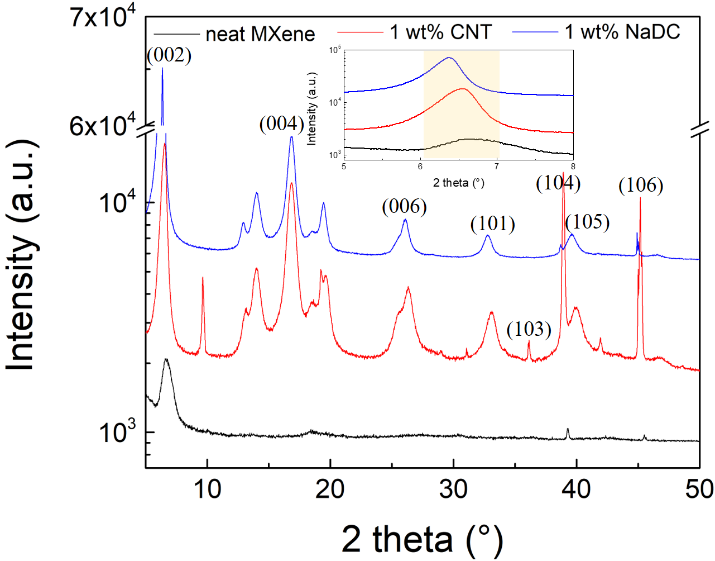


**Fig. S19** XRD patterns of neat Ti_3_C_2_T_x_ processed by 10000 rpm centrifugation and its nanocomposites with 1 wt% SWCNT and 1 wt% NaDC, respectively


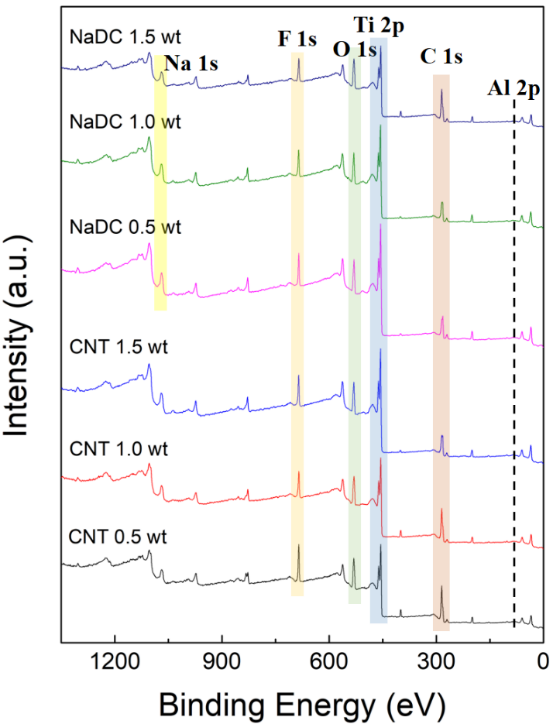


**Fig. S20** XPS profiles of Ti_3_C_2_T_x_ nanocomposites with various contents of SWCNT and NaDC


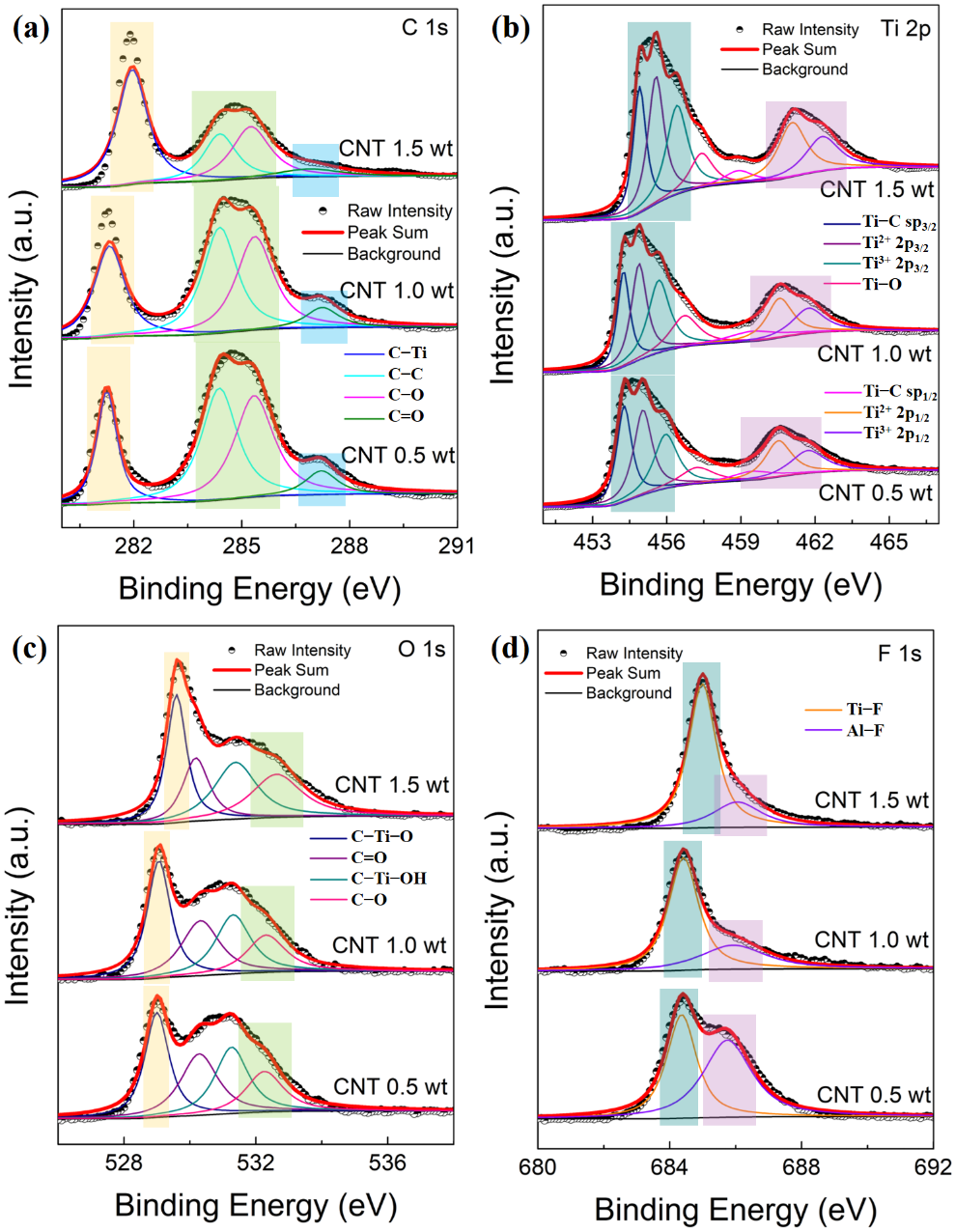


**Fig. S21** XPS profiles of (**a**) C 1*s*, (**b**) Ti 2*p*, (**c**) O 1*s* and (**d**) F 1*s* for Ti_3_C_2_T_x_ nanocomposites with various contents of SWCNT


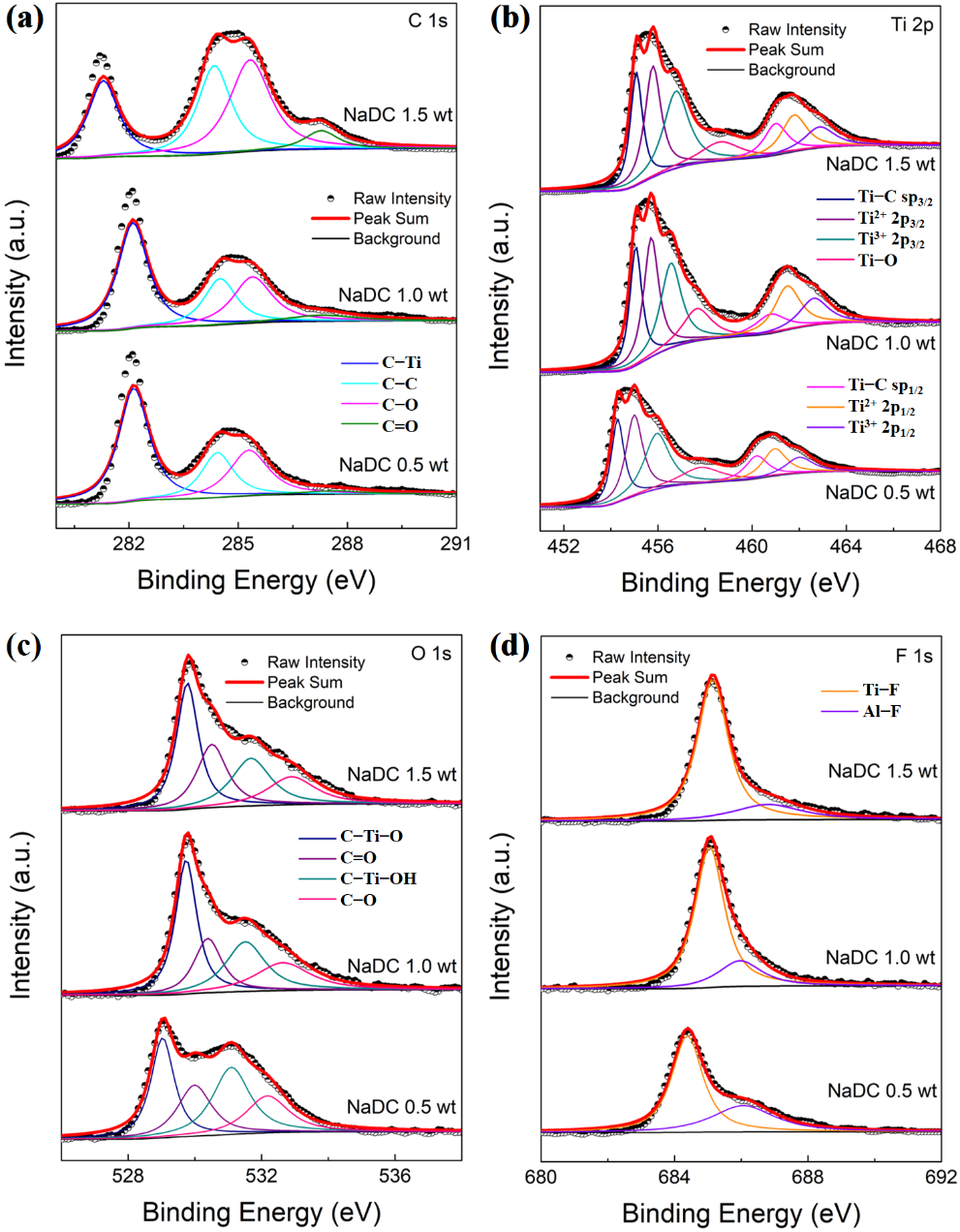


**Fig. S22** XPS profiles of (**a**) C 1*s*, (**b**) Ti 2*p*, (**c**) O 1*s* and (**d**) F 1*s* for Ti_3_C_2_T_x_ nanocomposites with various contents of NaDC


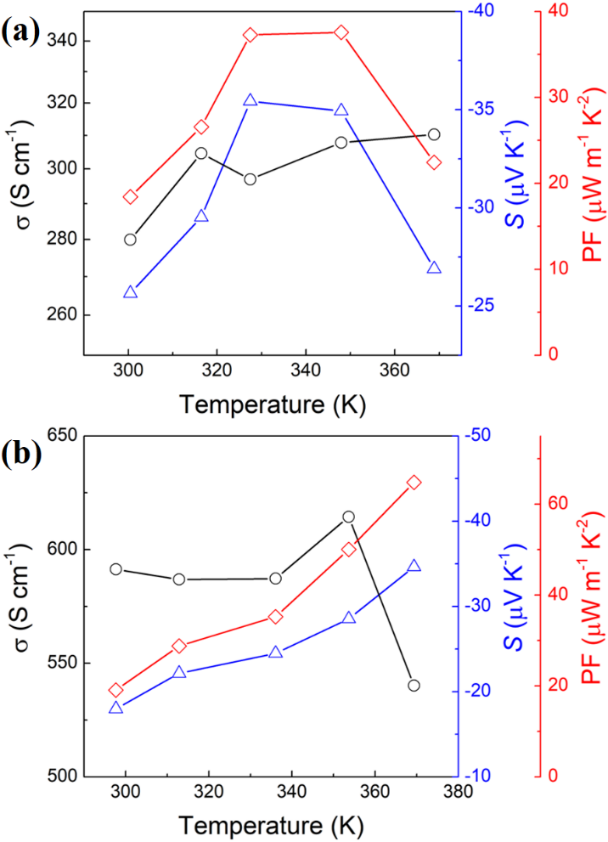


**Fig. S23** Temperature dependent TE performance of nanocomposites consisting of Ti_3_C_2_T_x_ MXene processed at 10000 rpm centrifugation with 1 wt% (**a**) SWCNT and (**b**) NaDC

**Table S1** Comparison of room-temperature TE performance of MXene films with literature

| **Material** | ***S* (μV K^−1^)** | ***σ* (S cm^−1^)** | **PF (μW m^−1^ K^−2^)** | **References** |
| --- | --- | --- | --- | --- |
| Ti_3_C_2_T_x_ | −8.2 ± 0.5 | 20652 ± 512 | 138.2 ± 17.7 | This work |
| Ti_3_C_2_T_x_ | −5.5 | 2200 | 6.65 | [S1] |
| K-Ti_3_C_2_T_x_ | −16.5 | 1652 | 44.98 |  |
| Mo_2_TiC_2_T_x_ | ~−22 | ~1500 | ~72.6 | [S2] |
| Nb_2_CT_x_ | 30 | 121.3 | 11.06 | [S3] |
| Mo_2_TiC_2_T_x_ | −27 | 169.5 | 13.26 |  |
| Ti_3_C_2_T_x_ | −5 | ~8000 | ~20 |  |

**Table S2** Comparison of the output performance of MXene-based TE device with literature

| **Materials** | **No. of legs** | **ΔT**  **(K)** | ***V*_T_**  **(mV)** | ***P*_max_**  **(nW)** | **References** |
| --- | --- | --- | --- | --- | --- |
| n-leg: Ti_3_C_2_T_x_ | 5 | 30 | 1.21 | 39.64 | This work |
| p-leg: Mo_2_CT_x_  n-leg: Mo_2_TiC_2_T_x_ | 4 | 25 | 3.43 | 22.7 | [S4] |
| p-leg: Nb_2_CT_x_  n-leg: Mo_2_TiC_2_T_x_ | 40 | 30 | 35.1 | 33.5 | [S3] |
| p-leg: Mo_2_C  n-leg: Mo_2_Ti_2_C_3_ | 400 | 5.4 | 399.9 | 93.6 | [S5] |

**Supplementary References**

1. P. Liu, W. Ding, J. Liu, L. Shen, F. Jiang et al., Surface termination modification on high-conductivity MXene film for energy conversion. J. Alloys Compd. **829**, 154634 (2020). <https://doi.org/10.1016/j.jallcom.2020.154634>
2. H. Kim, B. Anasori, Y. Gogotsi, H. N. Alshareef, Thermoelectric properties of two-dimensional molybdenum-based MXenes. Chem. Mater. **29**, 6472–6479 (2017). <https://doi.org/10.1021/acs.chemmater.7b02056>
3. D. Huang, H. Kim, G. Zou, X. Xu, Y. Zhu et al., All-MXene thermoelectric nanogenerator. Mater. Today Energy **29**, 101129 (2022). <https://doi.org/10.1016/j.mtener.2022.101129>
4. Z. Du, K. Deng, F. Wang, X. Liu, B. Jiang et al., Undelaminated multilayer MXenes for block thermoelectric generators. J. Mater. Chem. A **12**, 16648−16656 (2024). <https://doi.org/10.1039/D4TA01940G>
5. T. Park, K. Cho, S. Kim, Thin-film thermoelectric generators comprising molybdenum-based MXenes pn modules. Adv. Mater. Technol. **6**, 2100590 (2021). <https://doi.org/10.1002/admt.202100590>
